# Development and Identification of SSR Markers Associated with Starch Properties and β-Carotene Content in the Storage Root of Sweet Potato (*Ipomoea batatas* L.)

**DOI:** 10.3389/fpls.2016.00223

**Published:** 2016-03-02

**Authors:** Kai Zhang, Zhengdan Wu, Daobin Tang, Changwen Lv, Kai Luo, Yong Zhao, Xun Liu, Yuanxin Huang, Jichun Wang

**Affiliations:** ^1^College of Agronomy and Biotechnology, Southwest UniversityChongqing, China; ^2^Engineering Research Center of South Upland Agriculture, Ministry of Education, Southwest UniversityChongqing, China; ^3^Sweet Potato Engineering and Technology Research CenterChongqing, China

**Keywords:** amylose, β-carotene, marker development, marker-trait association, SSR, starch, sweet potato

## Abstract

Sweet potato (*Ipomoea batatas* L.) is a nutritious food crop and, based on the high starch content of its storage root, a potential bioethanol feedstock. Enhancing the nutritional value and starch quantity of storage roots are important goals of sweet potato breeding programs aimed at developing improved varieties for direct consumption, processing, and industrial uses. However, developing improved lines of sweet potato is challenging due to the genetic complexity of this plant and the lack of genome information. Short sequence repeat (SSR) markers are powerful molecular tools for tracking important loci in crops and for molecular-based breeding strategies; however, few SSR markers and marker-trait associations have hitherto been identified in sweet potato. In this study, we identified 1824 SSRs by using a *de novo* assembly of publicly available ESTs and mRNAs in sweet potato, and designed 1476 primer pairs based on SSR-containing sequences. We mapped 214 pairs of primers in a natural population comprised of 239 germplasms, and identified 1278 alleles with an average of 5.972 alleles per locus and a major allele frequency of 0.7702. Population structure analysis revealed two subpopulations in this panel of germplasms, and phenotypic characterization demonstrated that this panel is suitable for association mapping of starch-related traits. We identified 32, 16, and 17 SSR markers associated with starch content, β-carotene content, and starch composition in the storage root, respectively, using association analysis and further evaluation of a subset of sweet potato genotypes with various characteristics. The SSR markers identified here can be used to select varieties with desired traits and to investigate the genetic mechanism underlying starch and carotenoid formation in the starchy roots of sweet potato.

## Introduction

Sweet potato (*Ipomoea batatas* (L.) Lam.) is an important food crop cultivated in over 100 countries (Gao et al., 2000; Huang and Sun, 2000; Hu et al., 2003). Its high yielding potential and adaptability to a wide range of environmental conditions make it an important staple and food security crop in many areas of the world, especially in developing countries (Schafleitner et al., 2010; Wang Z. et al., 2011). Sweet potatoes are nutritious and contain high levels of dietary fiber (Vimala et al., 2011), minerals (such as iron), and vitamins A, B, and C (Low et al., 2007; Nedunchezhiyan et al., 2010). As a universal crop, sweet potato is cultivated for general consumption (fresh roots and leaves), is processed into livestock feed (Nedunchezhiyan et al., 2012), is grown as ornamental vines, and is an industrial raw material used to produce flour, candy, natural pigment, and a variety of starch-based industrial products (Hu et al., 2003; Nedunchezhiyan et al., 2010, 2012).

Sweet potato is mainly grown for its starchy root (Nedunchezhiyan et al., 2010), which is an important agricultural and biological organ (Wang et al., 2010). Its high starch content renders sweet potato a cheap and strong candidate source for biofuel production (Ziska et al., 2009; Nedunchezhiyan et al., 2012). The dry matter content of the storage root is essential for post harvest processing (Nedunchezhiyan et al., 2010), and the composition of starch in the storage root, particularly the ratio of amylose to amylopectin, also influences the physicochemical properties of starch (Hamada et al., 2006; Zhou et al., 2015) and ethanol yield (Nedunchezhiyan et al., 2012). Furthermore, the storage root of sweet potato is considered an excellent source of health-promoting compounds, such as β-carotene and antioxidative anthocyanins (Bovell-Benjamin, 2007). The high level of β-carotene (a precursor of vitamin A) (Low et al., 2007; Teow et al., 2007) in orange-fleshed sweet potato (OFSP, Low et al., 2007; Burri, 2011) may help to mitigate vitamin A deficiency-related blindness and maternal mortality in many developing countries (Schafleitner et al., 2010; Vimala et al., 2011; Wang Z. et al., 2011; Agili et al., 2012; Mitra, 2012). Developing sweet potato varieties with high levels of dry matter content, starch, and β-carotene is an important goal of breeding programs aimed at generating improved varieties for direct consumption, processing, and industrial applications (Nedunchezhiyan et al., 2012).

Despite its economic importance, sweet potato is still regarded as a fringe crop, with several factors blocking its development and utilization. Firstly, breeding is limited by its sterility and cross-incompatibility (Kitahara et al., 2007). Secondly, reliable phenotypic selection requires trials that take place in multiple locations and over many years. Furthermore, studies of the mechanisms underlying the formation of important traits are hampered by the lack of genome information. Therefore, efforts to breed superior varieties of sweet potato that combine high yield with root traits optimized for various end uses have had limited success. Sweet potatoes are mainly vegetatively propagated, which may accelerate the degeneration of cultivars (Adikini et al., 2015; Gibson and Kreuze, 2015). Rapid breeding practices are desired for shortening the breeding cycle of new varieties.

Breeding sweet potato varieties with desirable traits could be facilitated and accelerated through marker-assisted selection (MAS, Schmitt et al., 2010; Miah et al., 2013). Using DNA-based markers that are diagnostic of alleles of genes that underlie favorable traits early in the breeding cycle would drastically reduce the number of clones that must be propagated and evaluated in field trials (Li et al., 2013), thus improving the chances of developing new lines. Genetic markers linked to target traits therefore need to be identified. Several strategies could be used to identify such markers, such as quantitative trait locus (QTL) identification, differential gene expression analysis, or association mapping (Schmitt et al., 2010). Given that cultivated sweet potato is a hexaploid (2*n* = 6*x* = 90) outcrossing crop with a large genome size (2205 Mb) and a high degree of heterozygosity, genetic analysis, and QTL identification are challenging.

Developing molecular markers that facilitate the analysis of genetic traits is essential for MAS and crop improvement. Simple sequence repeats (SSRs; also called microsatellites) are useful genetic markers in many organisms. Due to their co-dominant and highly polymorphic nature, their even distribution throughout the genome, and their relative ease of development from enriched genomic libraries and expressed sequence tag (EST) collections (Collard and MacKill, 2008; Miah et al., 2013), SSRs are the marker of choice in linkage and genetic map construction (Varshney et al., 2009; Bindler et al., 2011; El-Rodeny et al., 2014), genetic diversity and population structure analysis (Barkley et al., 2006; Zhang et al., 2011; Chen et al., 2012; Xiao et al., 2012; Yoon et al., 2012), germplasm identification (Hong et al., 2015), and QTL mapping (Collard and MacKill, 2008; Varshney et al., 2009).

At least 2000 SSRs have been developed as potential molecular markers in sweet potato through different approaches, including screening of genomic libraries (Buteler et al., 1999; Hu et al., 2004), mining of publicly available EST sequences (Hu et al., 2004; Schafleitner et al., 2010; Wang Z. et al., 2011), and mining of EST sequences derived from high-throughput RNA sequencing analyses using Illumina HiSeq 2000 sequencing or 454 pyrosequencing (Schafleitner et al., 2010; Wang et al., 2010; Wang Z. et al., 2011; Tao et al., 2012; Xie et al., 2012). Two classes of SSRs exist; those of genomic origin and those of EST origin. Most SSR markers in sweet potato were designed based on EST-SSR-containing sequences. However, only a subset of these markers has been proved could successfully amplified or exhibited polymorphisms between several different sweet potato genotypes (Buteler et al., 1999; Hu et al., 2004; Schafleitner et al., 2010; Wang et al., 2010; Wang Z. et al., 2011). The number and availability of SSR markers were limited when compared to other crops. Association analysis between these SSR markers and traits across diverse genetic backgrounds has not been performed. Despite their limitations, SSR markers have been used in genetic diversity analyses (Zhang et al., 2001; Hwang et al., 2002; Gichuru et al., 2006; Veasey et al., 2008; Karuri et al., 2009; Yada et al., 2010; Tumwegamire et al., 2011) and in studies of the origin and dispersal of sweet potato (Roullier et al., 2011).

Association mapping (also known as linkage disequilibrium mapping) is a powerful approach for identifying genotype (marker)-phenotype (trait) correlations within a diverse collection of germplasms or breeding materials (Myles et al., 2009; Wang M. L. et al., 2011). Marker combinations associated with target traits can be identified using a natural population and further validated by testing whether the expected phenotypic effects are reproducible in populations that differ from the one in which the marker-trait association was originally identified (Li et al., 2013).

In this study, we developed SSR markers in sweet potato and examined whether these markers were associated with important quality traits, such as dry matter content, β-carotene content, and starch content and composition of the storage root, in a set of 239 sweet potato accessions. The objectives of this study were to (1) design novel PCR primer pairs from newly assembled sweet potato sequences; (2) determine whether the employed SSR markers are associated with the evaluated quality traits in this diverse collection, and (3) confirm that these identified markers amplified distinct bands in diverse sweet potato genotypes. This is the first report of an SSR marker-trait association analysis of multiple agronomic quality traits in sweet potato.

## Materials and methods

### Plant materials

A population of 239 genotypes of sweet potato consisting of standard cultivars, wild varieties, farmer varieties, landraces, and breeding materials (breeding clones) was used for association mapping. These 239 genotypes have a wide range of morphological types and are derived from various geographical origins, and were selected from more than 500 germplasms collected from prominent sweet potato producers in China and other countries, on the basis of cluster analysis and phenotypic selection. An overview of accessions and their origins is presented in Supplementary Material 1. Stem cuttings of all genotypes were planted in June of 2011–2013 and grown under natural conditions in Beibei, Chongqing, China, in the growing seasons of 2011, 2012, and 2013. The dry matter, starch, amylose, amylopectin, and β-carotene content of storage roots were evaluated in each of the 3 years.

### Determination of quality characteristics

#### Dry matter, starch, and β-carotene content measurement

Storage roots were harvested from three to five plants per genotype and starch traits (including starch, amylose, and amylopectin content) and β-carotene content were estimated. Dry matter content was measured using a similar method as described previously (Cervantes-Flores et al., 2011). The total starch content of storage roots was calculated based on the dry matter content using a reported conversion formula (Wang et al., 1989). For each genotype, β-carotene was extracted from approximately 1 g of freshly harvested storage root using acetone, as described by Ma et al. (2009). The concentration of β-carotene was determined by comparison with an external standard curve, generated using the absorption coefficient of pure β-carotene (Sigma-Aldrich, USA) on a Model 752 UV–visible spectrophotometer (Modern Science Optical Instrument Co. Ltd, Shanghai, China), at a wavelength of 454 nm.

#### Starch composition measurement

For quantitative analysis of the amylose and amylopectin content, the total starch was extracted and stained with acetic acid and I_2_ solution [0.4% (w/v) KI/0.02% (w/v) I_2_] for 10 min following an established method (Huang et al., 2010), and the amylose and amylopectin content of the total starch were then measured using a colorimetric method with a Model 752 UV–visible spectrophotometer (Modern Science Optical Instrument Co. Ltd, Shanghai, China), at a wavelength of 630 and 548 nm, respectively. The concentrations were quantified using external standard curves prepared with pure amylose from potato (Sigma-Aldrich, Gillingham, UK) and pure amylopectin from maize (Fluka, Sigma-Aldrich) as standard samples, respectively.

The amylose and amylopectin content of the storage roots were first calculated as a percentage of dry weight of total starch. To ensure accuracy, the ratio of amylose content to amylopectin content (denoted here as A/P) was also calculated and used as a marker of starch composition.

The assessment of dry matter content, starch content, β-carotene content, and starch composition were carried out in triplicate in each experimental year. Statistical analysis of trait data was performed with SPSS (version 20.0; RRID: rid_000042).

### SSR marker development

#### Data mining and transcript assembly

Pair-end, single-end, and 454 sequencing reads and publicly available EST and mRNA sequences were obtained from the EST database at NCBI (http://www.ncbi.nlm.nih.gov/; RRID:nlx_62971) using the ENTREZ search tool and sweet potato root transcriptome data (http://batata.agri.gov.il/), and were *de novo* assembled in this study. Pair-end reads were *de novo* assembled using Trinity (Grabherr et al., 2011; RRID:OMICS_01327). CLC Genomics Workbench 7.5.1 (CLC Bio, Aarhus, Denmark; RRID:OMICS_01124) was used for *de novo* assembly of the pair-end reads, single-end reads, and 454 sequences. To enhance the efficiency of assembly and to obtain the longest contigs, all the sequences assembled using Trinity and CLC Genomics Workbench 7.5.1 were finally assembled together with publicly available ESTs and mRNAs using CLC Genomics Workbench 7.5.1.

#### Analysis of SSRs and primer design

Perl script MISA software (MISA http://pgrc.ipk-gatersleben.de/misa/; RRID:OMICS_00110) was used to mine microsatellites from the assembled sequences. Sequences with at least nine repetitions for dinucleotides, six repetitions for trinucleotides, and five repetitions for tetra-, penta-, and hexa-nucleotides, excluding the polyA and polyT repeat, were identified. Dinucleotide repeats such as AT/TA and CT/GA were treated as the same type of repeat motif.

Primer Premier 7.0 software (PREMIER Biosoft International, Palo Alto, CA) was used to design appropriate primers from the flanking sequences, based on the following criteria: primer length of 18–22 bp (optimally 20 bp), GC content of 40–60%, annealing temperature (Tm) of 50–60°C (with the Tm of forward and reverse primers differing by no more than 4°C), and expected amplified product size of 100–400 bp. Primers were synthesized by Invitrogen Biological Engineering Technology & Services Co., Ltd (Shanghai, China).

### DNA extraction and SSR marker assays

Genomic DNA was extracted from fresh young leaves following the CTAB protocol (Kim and Hamada, 2005) with slight modification. The concentration and quality of extracted DNA were visually examined using GoldView (SBS Genetech, Beijing)-stained 1% agarose gels and assessed using a NanoDrop 2000 UV–Vis spectrophotometer (Thermo Fisher scientific, USA). Among the developed SSR markers, 306 pairs of primers were initially screened for their ability to detect polymorphisms among eight sweet potato accessions (i.e., D01414, Yushu 33, Shangqiu 52-7, Chaoshu No. 1, Xushu 22, Ning 4-6, S1-5, and Fengshouhong). Markers exhibiting polymorphisms among these accessions were selected for further analysis of 239 genotypes. PCR reactions were carried out in a total volume of 10 μL containing 15 ng template DNA, 0.5 U *Taq* DNA polymerase (TransGen Biotech, Beijing), 0.20 mM dNTPs (Dingguo Biotech, Beijing), 1 μM of each primer, and 1.2 μL of 10 × PCR buffer (containing 20 mM mgSO_4_). PCR amplifications were performed in a 9700 Thermal Cycler (ABI, USA) under the following cycle profile: 5 min at 94°C; 35 cycles of 45 s at 94°C, 45 s at an annealing temperature of 55°C, and 1 min at 72°C; and a final elongation of 10 min. PCR products were analyzed on 10% polyacrylamide gel electrophoresis (PAGE) and visualized by silver staining. Band size was estimated using a 100-bp DNA ladder (TransGen Biotech, Beijing). Polymorphic bands were used to assign loci for each primer and scored as present (1) or absent (0).

### Genetic diversity estimation

The number of alleles, genetic diversity, major allele frequency, and polymorphism information content (PIC) were assessed using PowerMarker ver. 3.25 (Liu and Muse, 2005; RRID:nlx_154544). The genetic distances between each pair of genotypes were estimated by calculating the Nei's standard genetic distance (Nei, 1972) using PowerMarker based on the genotyping results generated using SSR markers. The genetic distance matrix, based on Nei's genetic distance, was computed and clustered by the neighbor-joining (NJ) algorithm using PowerMarker, and the resulting dendrogram was observed using MEGA ver. 4.0 (Tamura et al., 2007; RRID:nlx_156838). The mean genetic distance among tested germplasms was also calculated using MEGA ver. 4.0 (Tamura et al., 2007).

### Population structure analysis

The population structure of the 239 sweet potato genotypes was assessed using the model-based Bayesian clustering method implemented in STRUCTURE v2.3.4 (Hubisz et al., 2009; RRID:nlx_154662) and allelic data from the SSR markers. The number of subpopulations (*K*) was set from 1 to 20 based on models characterized by admixture and correlated allele frequencies. For each *K*, five runs were performed separately, with 100,000 Monte Carlo Markov Chain (MCMC) replicates carried out for each run after a burn-in period of 10,000 iterations. A *K*-value was selected when the estimate of Ln Pr (*X*|*K*) peaked in the range of 1–20 subpopulations. Since the distribution of Ln Pr (*X*|*K*) did not show a clear cut-off point for the true *K*-value, an *ad hoc* quantity Δ*K* was used to detect the numbers of subpopulations (Evanno et al., 2005). The run with the maximum likelihood value was selected to assign the membership coefficients (*Q*) to each genotype and to generate the subpopulation component *Q* matrix. The results from STRUCTURE were displayed with DISTRUCT 1.1 software (Rosenberg, 2004). The run with the maximum likelihood was applied to subdivide the genotypes into different subpopulations using a membership probability threshold of 0.70 and the maximum membership probability among subpopulations. Those genotypes with a membership probability of less than 0.70 were retained in the admixed group (AD).

### Association mapping

Association analysis between individual polymorphisms and phenotypic values was carried out with the general linear model (GLM) and mixed linear model (MLM) method (Yu et al., 2006) implemented in the TASSEL 5.0 software package (Bradbury et al., 2007; RRID:nlx_154674) available at http://www.maizegenetics.net/tassel. The relative kinship coefficients (*K*-matrix) among all pairs of genotypes were estimated from SSR marker data using the kinship matrix function in TASSEL. GLM analysis was performed using the population structure *Q* matrix generated by STRUCTURE (mentioned above), or without controlling for the population structure (the naive model), and MLM analysis was performed using the kinship *K* matrix incorporating or without the population structure *Q* matrix. The number of permutations was set at 1000 to obtain the permutation-based test (Anderson and Ter Braak, 2003) of marker significance and the experiment-wise *P*-value for marker significance. Quantile–quantile plots of each model were conducted using the ggplot2 program (http://had.co.nz/ggplot2/) in the R package (http://www.r-project.org/) (Wickham, 2009). The SSR allele is significantly associated with target traits when the *P*-value is less than 0.01. The R^2^-value (marker R^2^ obtained using TASSEL, which represents R^2^ for the marker after fitting other model terms) indicates the percentage of variance explained by the associated allele. Only markers with an allele frequency of 5% or greater were included in the association analysis.

### Verification of SSR markers

To confirm the effectiveness of the detected SSRs, markers identified by association mapping were further validated using diverse sweet potato cultivars with various storage root starch or β-carotene properties. Clean and reproducible amplicons generated in these cultivars were compared or further sequenced. All PCR amplifications and electrophoresis analyses for each SSR marker were repeated more than three times.

## Results

### Phenotypic characterization of 239 sweet potato genotypes

**Figure 1:**
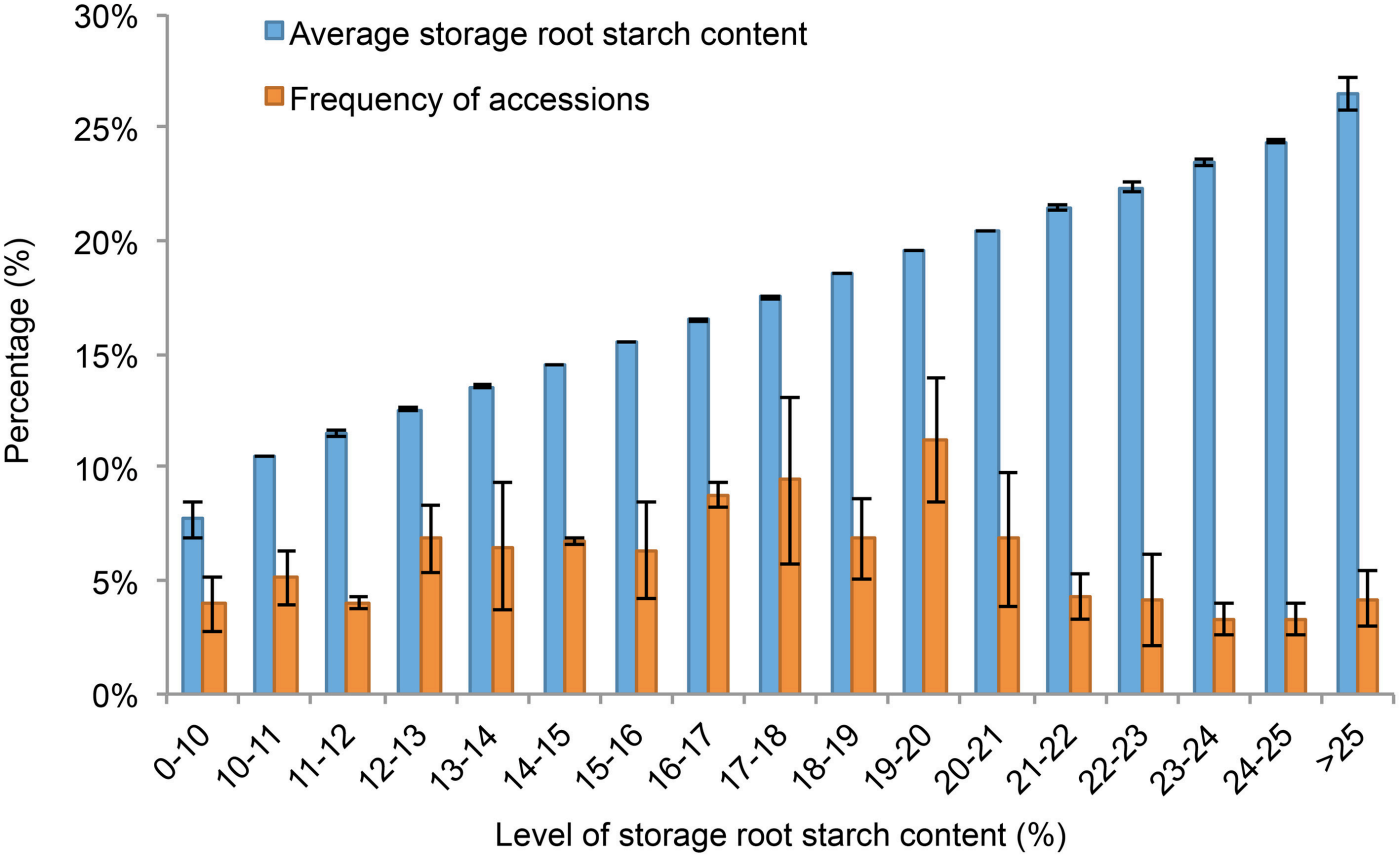
**The distribution of genotypes with specific ranges of storage root starch contents**. In 2011, 2012, and 2013, genotypes with a given range of storage root starch contents were selected and used for calculation of average storage root starch content and the frequency of accessions with this given range of storage root starch contents in the whole panel. Error bars indicate standard deviation from mean of calculated values obtained in the 3 experimental years.

**Figure 2:**
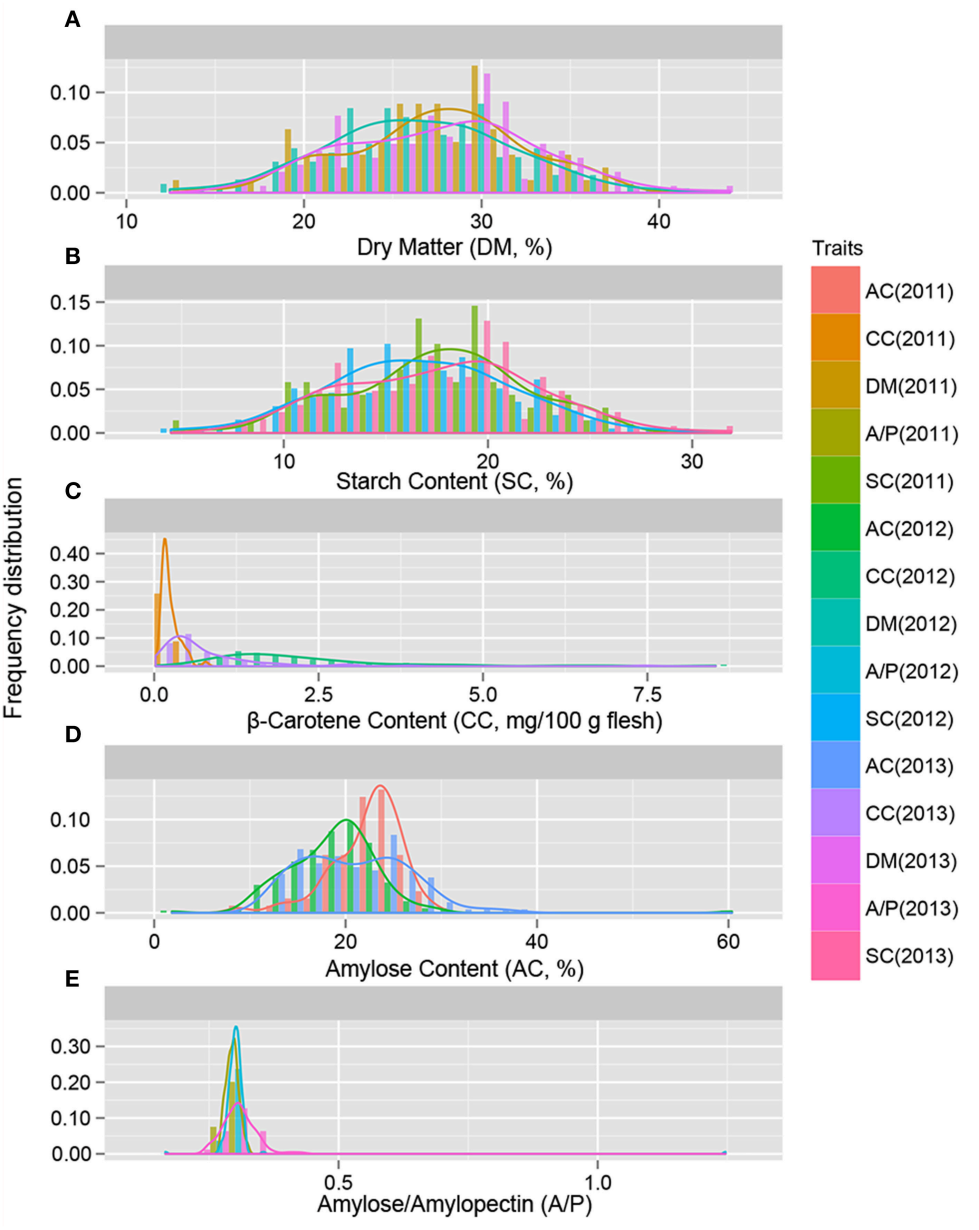
**The distribution of genotypes with the indicated level of storage root dry matter (A), starch content (B), β-carotene content (C), amylose content (D), and A/P (E)**. The traits were evaluated in 239 sweet potato genotypes in 2011, 2012, and 2013.

The descriptive statistics of quality traits are summarized in Table 1. The dry matter content of storage roots was estimated as ranging from 12.446 to 43.987% in the 239 sweet potato genotypes, and the starch content of storage root ranged from 4.476 to 31.899% (Table 1). As shown in Figures 1, 2A,B, the 239 sweet potato genotypes included varieties/lines with a range of dry matter and total starch contents in the storage roots, and the number of genotypes with each starch content range was relatively evenly distributed, indicating that this panel was suitable for association analysis for starch-related traits in sweet potato. For the starch composition-related traits, the amylose content of storage roots ranged from 7.280 to 35.512% (Table 1 and Figure 2D), and the ratio of amylose content to amylopectin content (A/P) ranged from 0.166 to 0.430 (Table 1 and Figure 2E). The highest β-carotene content observed amongst the 239 genotypes was 8.538 mg/100 g flesh weight, which was obtained in the OFSP variety Tainong 69 in the year 2012 (Table 1 and Figure 2C). For each genotype, One-way ANOVA analysis showed that there was no significant difference in the dry matter and starch contents of the storage root, but marked variation in starch composition and β-carotene content was noticed among the 3 years of the experiment (the difference was significant at *P* < 0.001; Table 1 and Figure 2).

**Table 1:** **Descriptive statistics of the traits evaluated in the 239 sweet potato genotypes**.

**Traits**		**Y2011**	**Y2012**	**Y2013**	**Total**	***P-value***
Dry matter (%)	Scope	12.941–36.876	12.446–41.098	15.788–43.987	12.446–43.987	0.082
	mean	26.931 ± 4.942	26.336 ± 5.126	27.594 ± 5.418	26.853 ± 5.199	
Starch content (%)	Scope	4.906–25.716	4.476–29.386	7.381–31.899	4.476–31.899	0.082
	mean	17.069 ± 4.296	16.552 ± 4.457	17.646 ± 4.711	17.001 ± 4.520	
Amylose content (%)	Scope	8.814–30.316	7.280–29.389	11.719–37.191	7.280–35.512	< 0.000^**^
	mean	21.853 ± 3.829	18.698 ± 5.206	20.980 ± 5.447	20.028 ± 5.220	
Amylose/Amylopectin	Scope	0.272–0.358	0.166–0.355	0.247–0.430	0.166–0.430	0.194
	mean	0.295 ± 0.012	0.303 ± 0.066	0.308 ± 0.031	0.303 ± 0.050	
β-carotene content (mg/100 g flesh)	Scope	0–0.7828	0–8.538	0–7.457	0–8.538	< 0.000^**^
	mean	0.237 ± 0.125	2.256 ± 1.491	0.772 ± 0.876	0.969 ± 1.206	

*P-value: significance of difference among traits data measured in 3 years derived from single factor analysis of variance tested by One-way ANOVA analysis and the LSD test using SPSS*.

** *P < 0.01; Y, No. Year*.

### Correlation between quality traits evaluated in storage roots of sweet potato

Correlation analysis demonstrated that the dry matter and starch content of storage roots correlated positively with the amylose content (two-sided test; *P* < 0.01; in 2011 and 2012). However, the starch content measured in 2013 was not significantly (*P* = 0.594) positively correlated with the amylose content, but was significantly (*P* = 0.028) negatively correlated with the A/P value, indicating that there was no distinct correlation between total starch content and starch composition in the storage roots of sweet potato. Although there was no distinct correlation between the starch and β-carotene content in 2012 and 2013, there was a significant negative correlation between starch and β-carotene content when the values obtained over the 3-year period were evaluated. A significant positive correlation between amylose content and A/P ratio (two-sided test; *P* < 0.01; in 2011 and 2012) was detected, but there was no distinct significant correlation between β-carotene content and starch composition-related traits (Table 2).

**Table 2:** **Pearson's correlation among five traits evaluated in the association population**.

**Traits**	**Dry matter**	**Starch content**	**β-carotene content**	**Amylose content**	**Amylose/Amylopectin**
Dry matter	1	1.000^**^/1.000^**^	−0.166^**^/0.380^**^	0.301^**^/0.582^**^	−0.108^*^/−0.114
Starch content	1.000^**^/1.000^**^	1	−0.166^**^/0.380^**^	0.301^**^/0.582^**^	−0.108^*^/−0.114
β−carotene content	***0.062**/*−*0.142*	***0.062**/*−*0.142*	1	−0.135^*^/0.298^**^	**0.044**/−0.139
Amylose content	0.386^**^/0.046	***0.386***g^**^/0.046	***0.006**/0.006*	1	0.360^**^/0.629^**^
Amylose/Amylopect	***−0.106**/*−0.186^*^	***−0.106**/*−0.186^*^	***−0.088**/0.175*	0.569^**^/0.202^*^	1

*Pearson's correlation among values of five traits obtained over the 3-year study period (the bold number above the diagonal) and the values obtained in 2011 (the regular number above the diagonal), 2012, and 2013 (the bold italicized number and italicized number on the lower diagonal, respectively)*.

* *P < 0.05*;

** *P < 0.01*.

### Data mining and transcript assembly

To design novel EST-SSR primer pairs from as many published microsatellites as possible, we collected reads resulting from high-throughput sequencing and publicly available ESTs and mRNAs and assembled them *de novo*. Two software tools and the following four steps were used in the assembly process: (1) EST sequences, including paired-end reads SRR063318 (Wang et al., 2010), SRR329935 (Xie et al., 2012), SRR331947 (Tao et al., 2012), and SRR335407 (Tao et al., 2013), from the sweet potato gene index established in previous studies, were first assembled using the Trinity platform, and 136,671 contigs resulting in 63,915 components were obtained, with an N50 size of 1244 and 1035 bp, respectively. (2) A total of 34,733 ESTs publicly available on May 8, 2011 and a total of 55,907 mRNA sequences and 23,406 ESTs available from May 8, 2011 to December 18, 2014 were collected. (3) CLC Genomics Workbench 7.5.1 was used for *de novo* assembly of the SRR063318, SRR329935, SRR331947, and SRR335407 pair-end reads, and the ERR297372, ERR297373, and ERR297371 single-end reads obtained by 454 sequencing (Firon et al., 2013), resulting in 90,751 transcripts, with an N50 size of 683 bp and total base pair count of 47.56 Mb. (4) To enhance the efficiency of assembly and obtain the longest contigs possible, the ESTs obtained using Trinity in step (1) and the 90,751 contigs obtained using CLC Genomics Workbench 7.5.1 in step (3) were assembled together with all publicly available ESTs and mRNAs collected in step (2) using CLC Genomics Workbench into 62,968 transcripts, with an N50 size of 1302 bp and total base pair count of 53.87 Mb (Table 3). The number of transcripts was similar to the 63,915 components obtained using Trinity in step (1), showing that the publicly available sweet potato database contains about 63,000 genes. The average transcript length was 833 bp long, which is longer than that of most transcripts identified in sweet potato transcriptomes assembled to date.

**Table 3:** **Summary of SSRs identified in this study**.

**Searching items**	**Numbers**
Total number of sequences examined	62,968
Total size of examined sequences (bp)	53,867,441
Total number of identified SSRs	1824
Number of SSR containing sequences	1631
Number of sequences containing more than one SSR	167
Number of SSRs present in compound formation	63
Di-nucleotide	604
Tri-nucleotide	1015
Tetra-nucleotide	154
Penta-nucleotide	27
Hexa-nucleotide	24

### SSR marker development and primer design

**Figure 3:**
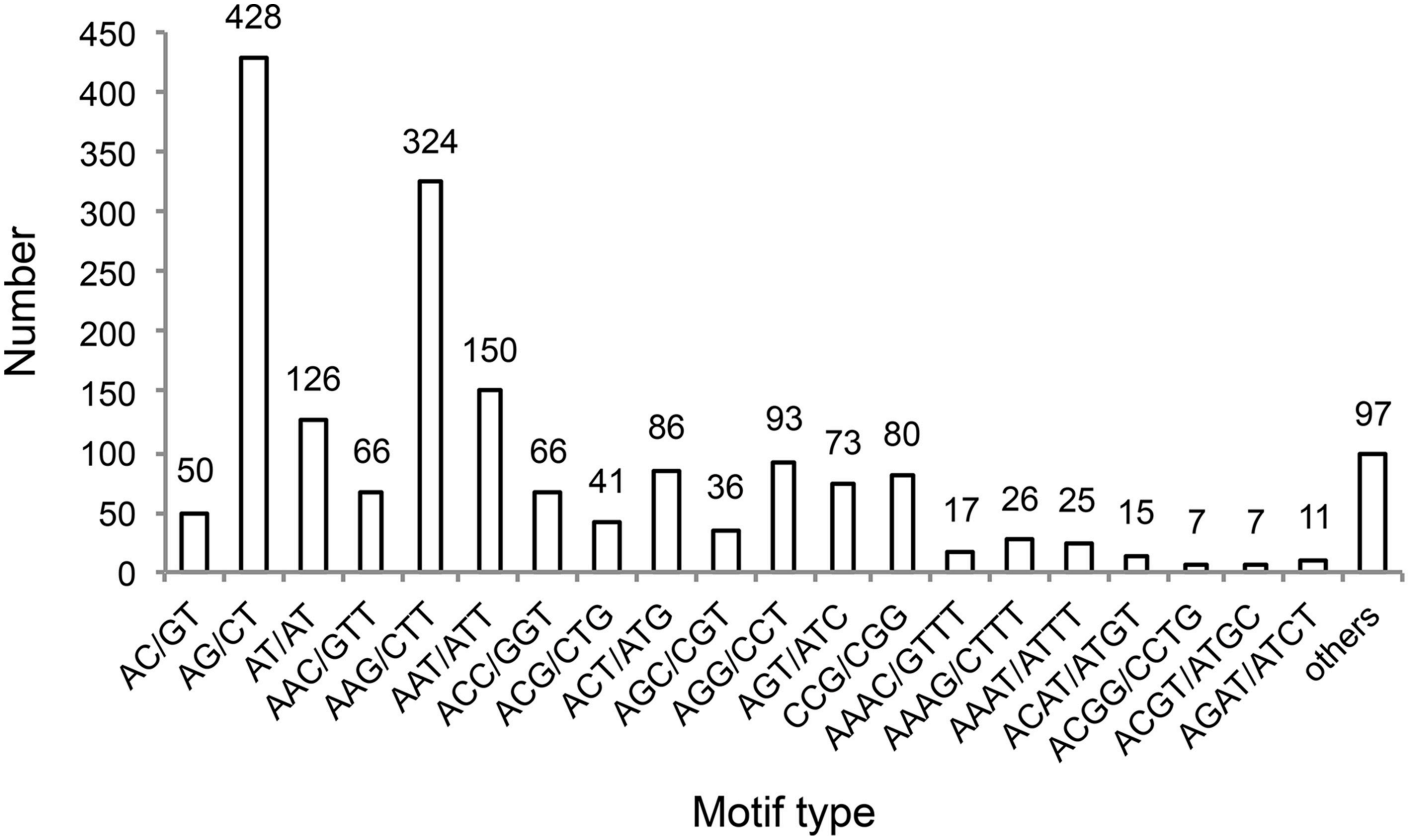
**Frequency of SSRs containing the indicated motif sequence types**.

We identified 1631 sequences containing SSRs, and found that 167 of these sequences contained more than one SSR (Table 3). Furthermore, we identified 1824 unique SSR motifs, containing 3, 10, 27, 16, and 18 types of di-, tri-, tetra-, penta-, and hexa-nucleotide repeats, respectively. SSR units of different type and length were not evenly distributed. Among the identified SSRs, the tri-, and di-nucleotide repeat motifs were the most abundant (1015, 55.647; 604, 33.114%, respectively), followed by tetra- (154, 8.443%), penta- (27, 1.480%), and hexa-nucleotide (24, 1.316%) repeat motifs (Table 3). The AG/CT dinucleotide repeat was the most abundant type of motif amongst the detected SSRs (428, 23.465% of all SSR motifs), followed by the trinucleotide repeat motif AAG/CTT (324, 17.763%). The remaining 72 types of motifs accounted for 58.772% of all SSR motifs (Figure 3). In addition, the most abundant SSR length was 18 bp (922 SSRs), accounting for 50.548% of the total SSRs, followed by 20 and 21 bp (239 and 193 SSRs, 13.103% and 10.581% of total SSRs, respectively). A maximum SSR length of 253 bp was observed.

Based on these SSR-containing sequences, 1476 pairs of high-quality SSR primers were designed using Primer Premier 7.0. Marker names for the 1476 primer pairs, the SSR motif, primer sequences, Tm (melting temperature), and expected product length are provided in Supplementary Material 2. Of these designed primers, 447, 808, 134, 22, 22, and 43 were for di-, tri-, tetra-, penta-, hexa-nucleotide, and compound formation repeats, respectively (Supplementary Material 2).

### SSR polymorphisms

After primarily testing the markers in eight sweet potato genotypes, we selected 214 primer pairs (69.935% of the initially tested 306 SSR primer pairs) that yielded polymorphic, sharp, and reproducible band patterns for further analysis in 239 sweet potato genotypes. A total of 1278 clean and distinct bands were revealed with a major allele frequency of 0.7702. Except for generating PCR products of the expected sizes, most primer pairs also amplified fragments that were larger or smaller than expected. The number of polymorphic bands obtained with each primer varied from 1 to 16, with a mean of 5.972, and most of the SSR markers produced 2–5 bands each. The mean polymorphism information content (PIC) was 0.2571, and the mean genetic diversity was 0.3122.

### Genetic relationships among sweet potato genotypes

SSR primers yielding polymorphic band patterns were also selected to evaluate genetic diversity amongst the 239 sweet potato genotypes. Using PCR amplification data based on the 214 SSR primers, the genetic distance matrix among all genotypes used in this study was calculated. The pairwise Nei's genetic distance of tested sweet potato genotypes detected by SSR markers ranged from 0.0509 to 0.9618, with a mean of 0.3834, indicating a high level of polymorphism in this sweet potato collection. The lowest genetic distance coefficient (0.0509) was identified between Fengshouhuang and Suyu No. 1, suggesting that these varieties were closely related. These two varieties originated from the same agro-ecological zone for sweet potato production in China (the Huang-Huai Basin spring and summer sweet potato region, Zhang et al., 2014), and from the same breeding parents (Nancy Hall and Okinawa No. 100, Supplementary Material 1). The highest Nei's genetic distance (0.9618) was found between Tainong No. 10 (a variety from Taiwan, China) and Kokei 14 (a variety introduced from Japan), implying that they were remotely related (Supplementary Material 3). Tainong No. 10 was obtained using two varieties introduced from the USA as parents. Similar to our genetic diversity analysis of sweet potato germplasm resources based on inter-simple sequence repeat (ISSR) markers (Zhang et al., 2014), cultivar Yushu No. 8 was found to be more remotely related to the other tested genotypes.

### Population structure distribution of 239 sweet potato germplasms

**Figure 4:**
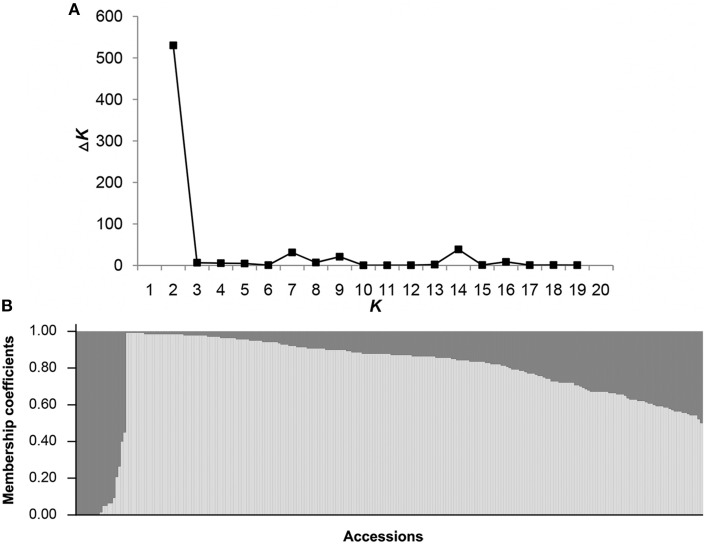
**Population structure of 239 sweet potato genotypes based on 214 SSR markers**. **(A)** STRUCTURE estimation of the number of subpopulations for *K* ranging from 1 to 20 by Δ*K*-values. **(B)** When *K* (the number of subpopulations) is 2, the 239 sweet potato genotypes were classified into two subpopulations, Pop 1 (light gray zone) and Pop 2 (dark gray zone).

The log likelihood revealed by STRUCTURE increased gradually from *K* = 1 to 20 and showed no obvious optimum (Supplementary Material 4). By contrast, the maximum *ad hoc* quantity Δ*K* was observed when *K* = 2 (Figure 4A), indicating the presence of two subpopulations (Pop 1 and Pop 2) in the entire population (Figure 4B). With membership probabilities of ≥ 0.70, 176 genotypes were assigned into Pop 1 and 17 genotypes into Pop 2. Furthermore, 46 genotypes with varying levels of membership shared between the two subpopulations were retained in the admixed group (AD) (Supplementary Material 5). Thus, there was no distinct subpopulation structure among the 239 genotypes. The respective *Q*-matrix outputs of the two subpopulations were used in the association analysis.

### Marker-trait association analysis and further verification of associated SSR markers

Excluding amplicons that were not of the expected length, a total of 887 polymorphic amplicons generated from 214 primer pairs were subjected to marker-trait association analysis. We performed association analyses using various mixed models (Yu et al., 2006; Myles et al., 2009), which with subpopulation membership percentages as fixed covariates or kinship as a random effect, including (1) naive model, without controlling for *Q* and *K*, (2) *Q* model, controlling for *Q*, (3) *Q*+*K* model, controlling for both *Q* and *K*, and (4) *K* model, controlling for *K* (Supplementary Material 6).

#### Identifying SSR markers associated with dry matter and starch content of storage roots

Sixty-three SSR markers had a much stronger association (*P* < 0.01) with the dry matter and starch content of the sweet potato storage root when tested using GLM after controlling the population structure, and 34 of these were also identified by MLM with kinship as a random effect (i.e., *Q*+*K* and *K* strategies). Markers SIP240 and SIP139 were only identified when the *Q*+*K* and *K* models were used. Markers SIP054, SIP068, SIP098, and SIP178 were only detected when using the naive model. Most of the SSR markers generated more than one association site. The tested *P*-values ranged from 3.70 × 10^−10^ (SIP029) to 0.00997 (SIP205), and the marker R^2^ ranged from 0.01592 (SIP285) to 0.28505 (SIP029). Allelic data for all SSR markers with significant association are presented in Table 4 and Supplementary Material 7. Twenty-four of the markers were found to be highly associated with trait data measured in 2 or 3 years.

**Table 4:** **Marker loci associated with dry matter and starch content of storage roots (*P* < 0.01)**.

**Marker**	**Model used**	**Based on phenotypic data Y2011**		**Based on phenotypic data Y2012**		**Based on phenotypic data Y2013**		**Based on phenotypic data 3 years**	
		***P*-value**	**R^2^**	***P*-value**	**R^2^**	***P*-value**	**R^2^**	***P*-value**	**R^2^**
SIP002	1–4	0.00546	0.08354					3.39E-04 5.58E-04	0.05346 0.04972
SIP029	1–4	0.00761	0.07734	9.37E-06	0.28505	0.00281	0.2169	1.95E-04	0.07096
		6.65E-04	0.12268					5.01E-04	0.06222
								3.70E-10	0.18804
								0.0086	0.03596
SIP098	1	4.73E-04	0.12894	0.00987	0.10758			5.54E-06	0.10372
		4.73E-04	0.12894	0.00987	0.10758			5.54E-06	0.10372
SIP125	1, 2			0.0032	0.11122			2.35E-05	0.07346
								5.23E-04	0.0502
								0.00452	0.034
SIP127	1, 2							0.00496	0.0333
								0.00808	0.02967
SIP137	1–4	3.62E-04	0.13386	0.00117	0.16491			3.27E-06	0.10851
		0.00236	0.09917	2.81E-04	0.20182			1.32E-04	0.07461
		5.84E-05	0.16682	0.00122	0.16364			7.86E-08	0.14187
		3.35E-04	0.13527	0.00265	0.14312			0.00115	0.05452
		0.00377	0.09046					1.15E-07	0.13852
								1.42E-04	0.07393
								1.02E-05	0.09818
SIP151	1–4	0.0014	0.14032	0.00174	0.04596	0.00977	0.04987	3.79E-06	0.0506
		0.0014	0.14032	0.00174	0.04596			3.79E-06	0.0506
								0.00132	0.02476
SIP160	1–4							0.00253	0.02155
								0.00315	0.02062
SIP173	1, 2			0.00289	0.04194			0.0064	0.01776
								1.91E-04	0.03299
SIP178	1							0.00785	0.01678
SIP183	1, 2	0.00783	0.09545	0.002	0.04463	0.00802	0.05205	1.32E-05	
		0.00112	0.13975	0.00302	0.04121			0.00705	
				0.00607	0.03538			6.78E-04	0.04432
								0.00321	0.01719
								3.56E-06	0.02719
								8.01E-05	0.02053
									0.05001
									0.03646
SIP187	1–4			7.32E-04	0.05283			0.00557	0.0218
								6.64E-04	0.03268
SIP192	1, 2							6.33E-04	0.02739
SIP195	1, 2	4.02E-04	0.16063	6.19E-04	0.05389	0.00881	0.05027	2.22E-07	0.06182
SIP200	1, 2	0.00727	0.09031					0.00148	0.02389
SIP202	1–4	0.00138	0.1296			3.26E-04	0.09535	2.32E-05	0.04253
		0.00754	0.09244					2.57E-04	0.03191
SIP213	1–4	8.63E-06	0.23066	0.0086	0.03247	6.63E-04	0.08407	2.74E-05	0.04122
		0.00133	0.12819	0.00384	0.03916			1.55E-06	0.05373
		0.00218	0.11767	0.00182	0.04544			0.00817	0.0166
				2.44E-04	0.06232			4.06E-04	0.02948
								1.35E-04	0.03425
								2.34E-04	0.03188
								8.72E-07	0.05624
SIP214	1–4			4.58E-04	0.05675			0.00324	0.0245
				4.81E-04	0.05634			0.00175	0.02763
				3.38E-04	0.05928			9.41E-04	0.03083
				4.01E-04	0.05786			2.26E-04	0.03819
				3.71E-04	0.05851			0.00248	0.02586
				0.00788	0.03303			0.00647	0.021
								0.00927	0.01919
								0.00351	0.0241
SIP222	1–4					0.00314	0.06444	2.59E-04	0.03187
						0.00799	0.05192	1.36E-04	0.03471
								0.00185	0.02326
SIP235	1–4			0.00369	0.0395	0.00996	0.04951	6.87E-04	0.03367
				0.00369	0.0395	0.00996	0.04951	6.87E-04	0.03367
								4.52E-05	0.04824
								4.52E-05	0.04824
SIP237	1–4	0.00307	0.04123	0.00206	0.04452			3.50E-06	0.05059
								0.00151	0.024
								0.00151	0.024
SIP240	3, 4					0.00802	0.05568		
SIP245	1–3	2.67E-04	0.15966					0.00525	0.01843
		0.00943	0.08474					0.00846	0.01649
								6.71E-04	0.02737
								0.00273	0.02131
SIP250	1–4	0.00581	0.10368	0.00109	0.04994			0.00539	0.02193
		0.00336	0.1164					0.00562	0.02188
								0.00702	0.02085
								2.31E-04	0.03835
SIP252	1–4	0.00318	0.11767	0.0065	0.03473			0.00561	0.0183
		0.00359	0.11488					0.00732	0.01716
		0.00484	0.10792						
SIP254	1–4	1.03E-04	0.19263					1.61E-04	0.03361
SIP261	1–4	0.00349	0.10755			0.00591	0.0558	0.00207	0.0225
						0.00439	0.05963	0.00316	0.02068
								0.00115	0.02502
SIP263	1–4	0.00218	0.11762					0.00379	0.01995
SIP265	1–4	8.27E-04	0.1383					0.00184	0.02307
SIP285	1–4	0.00276	0.12097	0.00879	0.03224	0.00797	0.05213	0.00128	0.02465
				9.41E-04	0.05088			0.00991	0.01592
				0.00983	0.03146			0.0048	0.01901
								4.12E-06	0.04988
SIP288	1–4			0.00659	0.03487			0.00502	0.02249
SIP300	1–4	0.00391	0.10988	0.00522	0.03656			5.68E-05	0.03816
				2.06E-04	0.06365				

*These markers were found to be associated with the corresponding traits when four models were used: 1, the naive model-without controlling for Q and K; 2, GLM used after controlling the population structure (Q); 3, MLM used after controlling the population structure (Q+K); and 4, MLM used without controlling for the population structure (K). When detected using various models, the P-values were obtained using model 2 or 3, which controlled for the population structure. The model information was the same as for Tables 5, 6, and Supplementary Materials 7, 9, 11*.

**Figure 5:**
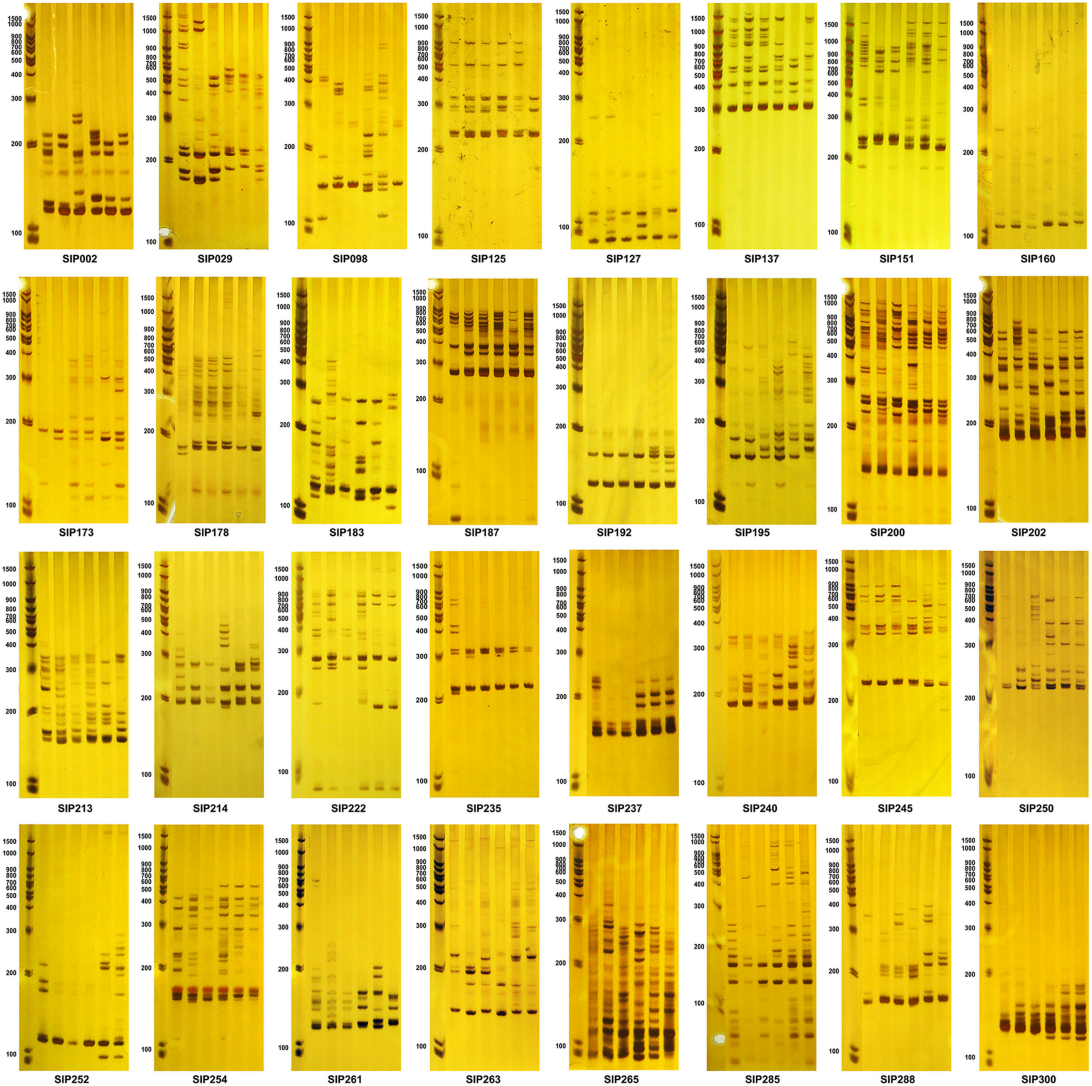
**Amplicons of the SSR markers associated with dry matter and starch content of the storage root**. The first lane of each figure is the molecular weight marker, and the next six lanes are amplicons amplified from DNA isolated from six accessions (from left to right: D01414, Yushu 33, Xushu 22, S1-5, Chaoshu No. 1, and Shangqiu 52-7), which had average storage root dry matter contents over the 3-year period of 37.387 ± 0.774, 34.469 ± 2.921, 27.956 ± 1.392, 21.088 ± 1.255, 19.032 ± 0.097, and 13.725 ± 1.803%, respectively, and average storage root starch contents over the 3-year period of 26.161 ± 0.673 (>25%), 23.623 ± 2.539 (20–25%), 17.961 ± 1.211 (15–20%), 11.989 ± 1.091 (10–15%), 10.201 ± 0.085 (about 10%), and 5.587 ± 1.568% (< 10%), respectively. All samples were genotyped at least in triplicate.

The 66 primers were used to further evaluate six diverse sweet potato genotypes, which were selected based on genotyping data measured over 3 years, and have different dry matter and starch content properties (Figure 5 and Supplementary Material 8). Thirty-two SSR markers (Table 4) that generated clean, reproducible, and distinctive bands for the six genotypes were identified as candidate markers that could distinguish between genotypes with different starch properties (Figure 5). Characteristic bands for genotypes with specific starch properties also could be found in the amplicons generated using these SSR markers. However, the other 34 SSR markers that were shown to be significantly associated with the starch content trait (Supplementary Material 7) also generated differential bands in the selected genotypes, but did not completely distinguish between genotypes with different range of starch content (Supplementary Material 8). It remains to be established whether these markers will also yield useful results in other sweet potato genotypes.

Moreover, we confirmed the association of five markers with dry matter and starch content in a hybrid population of 303 accessions generated using cultivars with high (Yushu 12) and medium levels of starch content (Luoxushu No. 9), i.e., SIP178 (*P* = 4.09 × 10^−4^), SIP200 (*P* = 0.00281), SIP222 (*P* = 0.00544), SIP261 (*P* = 0.00916), and SIP263 (*P* = 1.17 × 10^−4^, 0.00104 and 0.00133 for different loci), providing further evidence for the reproducibility of our present results obtained in the association analysis in different samples.

#### Association analysis of β-carotene content and SSR markers

Forty-four SSR markers showed a high association (*P* < 0.01) with the β-carotene content in the flesh of 239 sweet potato genotypes (*P*-value ranged from 2.70 × 10^−9^ to 0.00994). Markers SIP157, SIP209, SIP232, and SIP246 were only identified using MLM. Markers SIP193 and SIP250 were only detected using the naive model. Allelic data for SSR markers that revealed significant associations with β-carotene content are presented in Table 5 and Supplementary Material 9. Due to differences in values recorded in the 3 years of the experiment (Table 1), most of these markers were found to be associated with β-carotene content value measured in 1 year only. It should be noted that, because the β-carotene content was denoted as mg per 100 g flesh weight, these associations would also be affected by the dry matter content of the storage roots. Thus, among the identified markers, SIP29, SIP200, SIP213, and SIP250 were also associated with the dry matter content of the storage roots.

**Table 5:** **Marker loci associated with storage root β-carotene content (*P* < 0.01)**.

**Marker**	**Model used**	**Based on phenotypic data Y2011**		**Based on phenotypic data Y2012**		**Based on phenotypic data Y2013**		**Based on phenotypic data 3 years**	
		***P*-value**	**R^2^**	***P*-value**	**R^2^**	***P*-value**	**R^2^**	***P*-value**	**R^2^**
SIP019	1, 2	6.82E-05	0.19647						
SIP029	1, 2			7.50E-04	0.50356	0.00791	0.18013		
SIP126	1–4	0.00686	0.08204						
SIP147	1–4	0.00722	0.12794						
SIP150	1, 2					0.00823	0.05679	0.0017	0.05099
SIP175	1–4			5.83E-04	0.18887	1.41E-05	0.14941	2.70E-09	0.14062
SIP200	1–4	0.00768	0.13014			6.17E-04	0.09573	5.29E-04	0.04993
SIP203	1–4			0.00339	0.14092	0.00795	0.05868	8.86E-04	0.04623
				5.07E-04	0.19258	0.00887	0.05709	1.80E-05	0.07572
								4.44E-08	0.12037
								0.0064	0.03134
								1.10E-05	0.07942
								7.95E-05	0.0645
SIP211	1, 2							2.10E-04	0.05643
								1.58E-04	0.05856
SIP213	1, 2							2.32E-04	0.05617
SIP226	1, 2					0.00726	0.05949	9.42E-04	0.06042
						0.00726	0.05949	9.42E-04	0.06042
SIP228	1, 2					0.00643	0.06227	5.46E-04	0.05032
SIP246	3							0.00885	0.03978
SIP250	1							0.00299	0.03689
								0.0055	0.03246
SIP296	1–4					4.83E-04	0.09756	1.96E-05	0.09733
SIP302	1, 2			0.00468	0.12762			0.00579	0.03026

**Figure 6:**
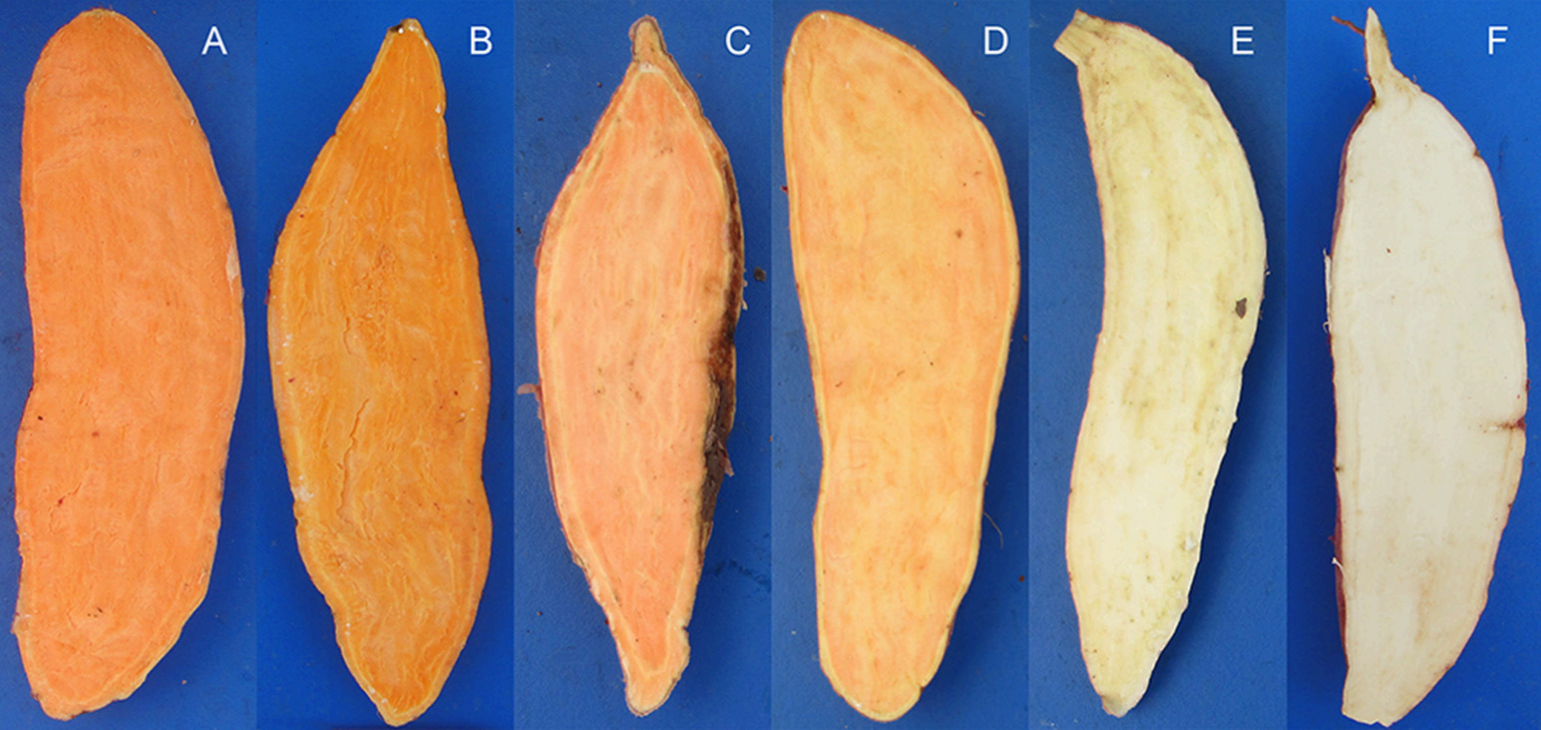
**The six sweet potato accessions used to validate the identified SSR markers associated with storage root β-carotene content**. The carotene content contributes to the light to dark orange color of the storage root flesh. **(A)**, Ning 4-6 (average β-carotene content over the 3-year period of 9.064 ± 1.133 mg/100 g flesh weight); **(B)**, Fengshouhong (6.249 ± 1.502 mg/100 g flesh weight); **(C)**, Erlangshao (4.510 ± 0.442 mg/100 g flesh weight); **(D)**, S1-5 (2.707 ± 0.263 mg/100 g flesh weight); **(E)**, Xichengshu 007 (0.132 ± 0.026 mg/100 g flesh weight); and **(F)** Zhe 147 (white-fleshed sweet potato with β-carotene levels that fell below the detection limit).

**Figure 7:**
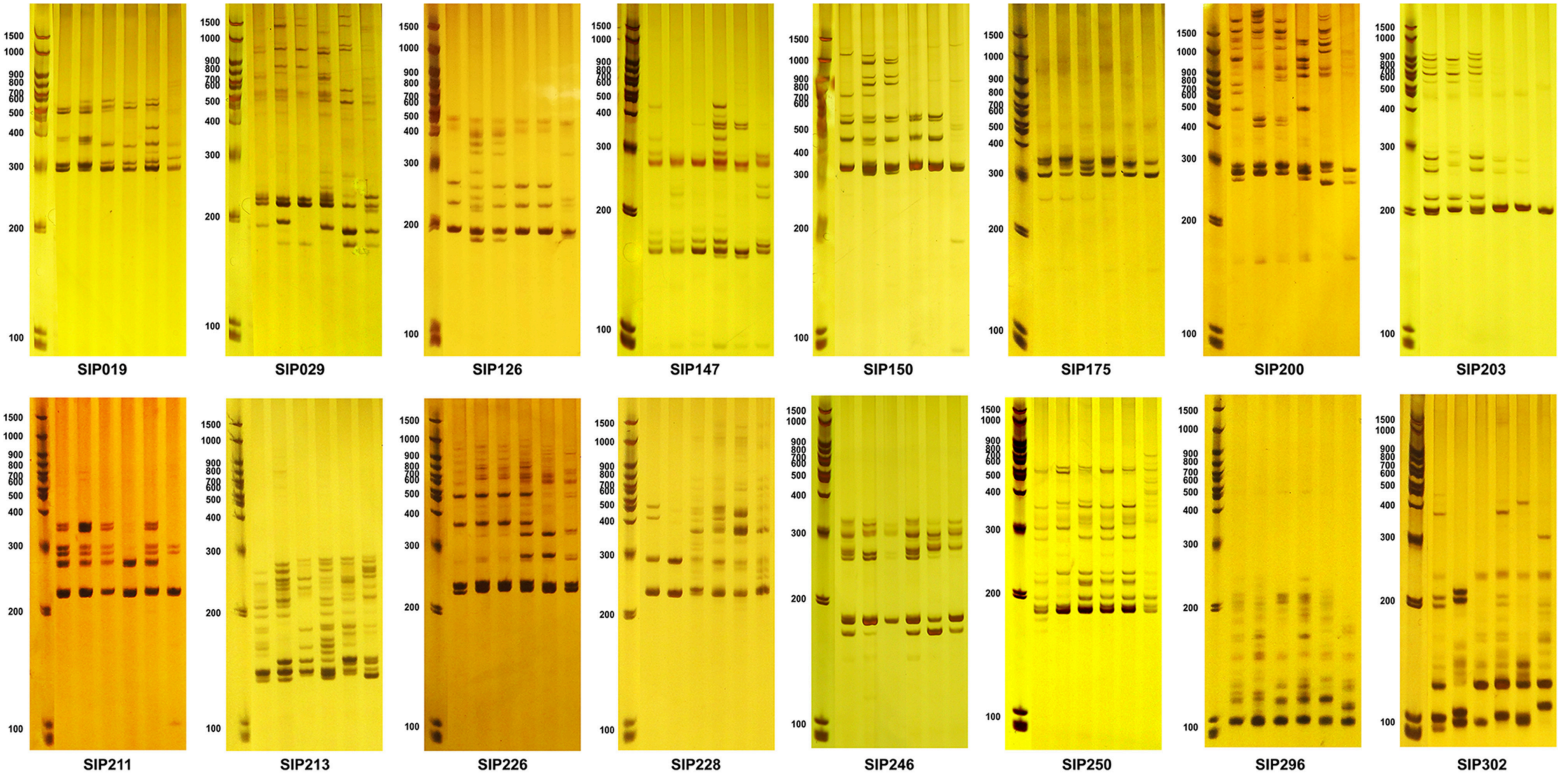
**Amplicons of SSR markers found to be associated with storage root β-carotene content in the six sweet potato accessions shown in Figure 6**. The first lane of each figure is the molecular weight marker, and the next six lanes are amplicons amplified from DNA isolated from the six accessions (from left to right): Ning 4-6, Fengshouhong, Erlangshao, S1-5, Xichengshu 007, and Zhe 147.

We mapped these markers using DNA extracted from six diverse sweet potato genotypes with different β-carotene properties (Figure 6). Sixteen SSR markers generated distinctive amplicons that could distinguish between the six selected genotypes (Table 5 and Figure 7). Compared to these markers, the other 28 SSR markers also showed differential bands in the selected genotypes, but showed less distinguishing effect on genotypes with specific β-carotene properties (Supplementary Materials 9, 10).

#### Association analysis of SSR markers and starch composition-related traits

Fifty-four SSR markers were found to have a significant association (*P* < 0.01) with starch composition-related traits, including the amylose content and A/P of the storage root, in the 239 sweet potato genotypes (Table 6 and Supplementary Material 11). Twenty-five of these SSR markers could be identified when analyzed using all the four models, and the tested *P*-value ranged from 1.53 × 10^−25^ (marker SIP215, associated with A/P) to 0.00999 (marker SIP004, associated with amylose content). Twelve markers were only identified when MLM (*Q*+*K* and *K* model) were used, which tested *P*-value ranged from 2.52 × 10^−4^ (marker SIP279, associated with A/P) to 0.00928 (marker SIP126, associated with amylose content). Marker SIP188, SIP195, and SIP203 were only identified when GLM was used (*P*-value from 2.67 × 10^−4^ to 0.0096, all associated with amylose content). However, 14 markers were only identified using the naive model, and the tested *P*-value ranged from 4.61 × 10^−4^ (marker SIP106, associated with amylose content) to 0.00989 (marker SIP110, associated with amylose content). Allelic data for all SSR markers with significant association are presented in Table 6 and Supplementary Material 11. Due to differences in amylose content and A/P-value of sweet potato genotypes measured in the 3 years (Table 1), most of these markers were found to be associated with starch composition-related traits measured in a single year. Among the identified markers, SIP125, SIP137, and SIP151 were also associated with the dry matter and starch content of storage roots.

**Table 6:** **Marker loci associated with storage root starch composition (*P* < 0.01)**.

**Marker**	**Model used**	**Based on phenotypic data Y2011**			**Based on phenotypic data Y2012**			**Based on phenotypic data Y2013**			**Based on phenotypic data 3 years**		
		**Trait**	***P*-value**	**R^2^**	**Trait**	***P*-value**	**R^2^**	**Trait**	***P*-value**	**R^2^**	**Trait**	***P*-value**	**R^2^**
SIP003	1	11A/P	0.00774	0.08745									
SIP004	1–4	11A/P	7.43E-04	0.13654	12A/P	0.00123	0.1561	13A	2.29E-04	0.31054	A/P	1.51E-04	0.09102
		11A/P	0.00167	0.11973	12A	0.00205	0.14315				A	0.00999	0.03485
SIP025	1										A	0.00466	0.05502
											A	0.00533	0.05339
SIP031	1–4				12A/P	0.00568	0.13177	13A/P	0.00478	0.19588	A/P	8.90E-04	0.07081
SIP125	1–4	11A	0.00346	0.09616				13A/P	0.00607	0.22288	A	2.61E-04	0.08111
		11A	0.00311	0.0982							A	0.00116	0.0648
SIP132	1				12A/P	0.00742	0.12156				A	0.00365	0.05796
SIP137	1–4				12A	0.00877	0.10566				A	0.00123	0.07112
					12A	0.00104	0.16051				A	9.44E-04	0.07436
SIP150	1–4	11A	3.01E-04	0.18856	12A/P	0.00376	0.04239				A/P	7.07E-04	0.02888
SIP151	1	11A/P	0.00374	0.13173									
SIP157	3, 4										A/P	0.00369	0.02103
SIP171	1–4				12A	0.0018	0.049				A	0.00491	0.02035
											A/P	0.00532	0.02315
SIP188	1, 2	11A	2.67E-04	0.1712							A	8.28E-04	0.02803
		11A	0.00732	0.09717									
SIP203	1, 2										A	0.00806	0.01749
											A	0.0096	0.01672
SIP218	1	11A	0.00528	0.11704									
		11A	0.00417	0.12305									
SIP232	1–4	11A	0.00145	0.13575	12A/P	7.12E-05	0.08219						
SIP283	1–4	11A/P	1.02E-04	0.21453							A	0.00355	0.02126
SIP291	1–4	11A	0.00138	0.16305	12A	0.00921	0.03444	13A/P	0.00873	0.05332			

*A, amylose content; A/P, ratio of amylose to amylopectin content*.

**Figure 8:**
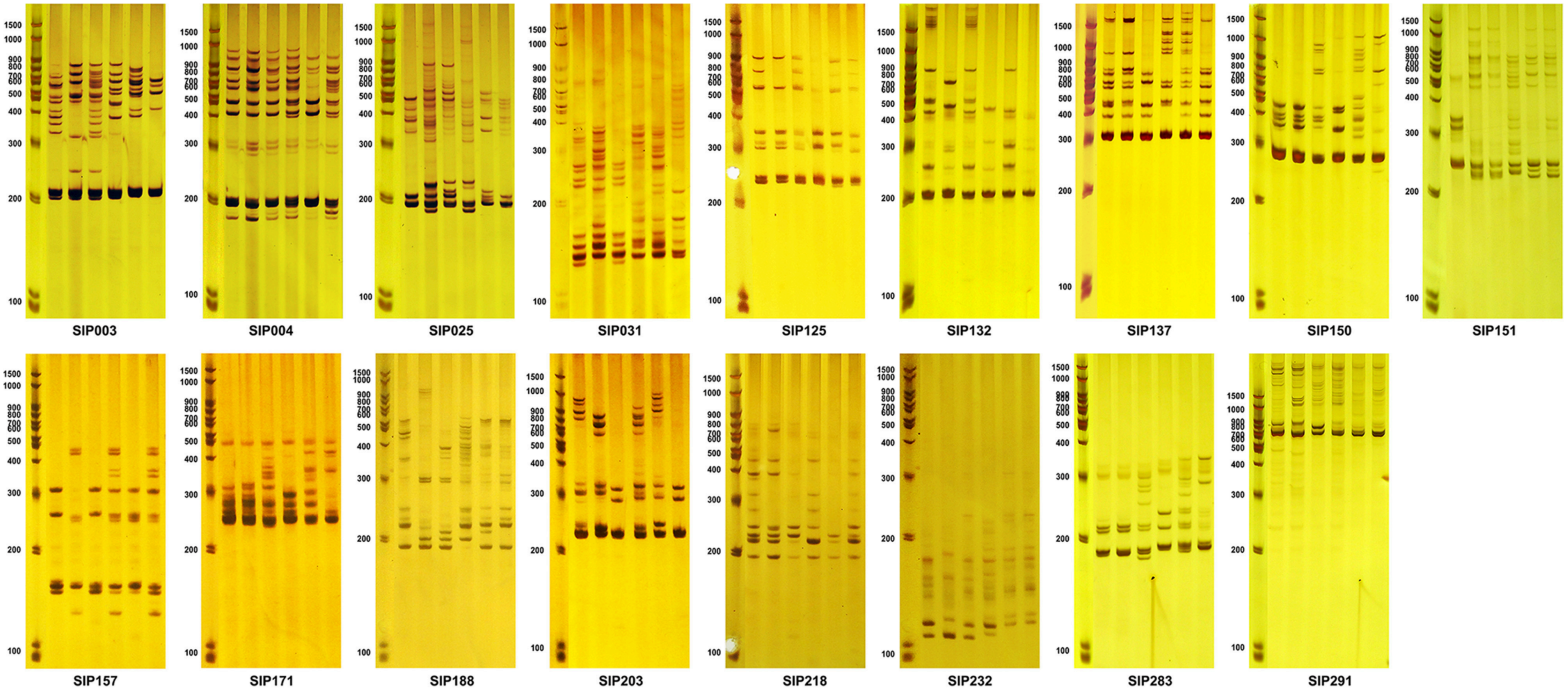
**Amplicons of SSR markers associated with the starch composition of the storage root**. The first lane of each figure is the molecular weight marker, and the next six lanes are amplicons amplified from DNA isolated from the six accessions. Lanes 2–4 (from left to right) are Mianfen No. 1, Nancy Hall, and 0929-106, which had high average storage root amylose contents over the 3-year period (28.465 ± 0.798, 26.081 ± 3.688, and 28.114 ± 1.776%, respectively), and high, medium, and low average storage root starch contents over the 3-year period (30.383 ± 3.713, 19.585 ± 0.231, and 10.857 ± 0.109%, respectively). Lanes 5–7 are Sanheshu, Jishu 52, and Wugonghong, respectively, with low average storage root amylose contents over the 3-year period (15.266 ± 1.656, 13.599 ± 1.206, and 12.076 ± 1.279%, respectively), and high, medium, and low average storage root starch contents over the 3-year period (20.210 ± 2.434, 18.336 ± 1.773, and 11.100 ± 0.824%, respectively).

In addition, six cultivars with high, medium, or low storage root starch or amylose content were selected to evaluate the amplicons generated using these identified markers (Figure 8 and Supplementary Material 12). When the 54 markers were further validated in the six diverse sweet potato cultivars, we identified 17 SSR markers that could distinguish between cultivars with high or low amylose content of the storage root (Table 6 and Figure 8). However, the other 37 SSR markers that were shown to be significantly associated with starch composition-related traits of storage root also generated differential bands in the selected cultivars, but did not distinguish between cultivars with high and low amylose content (Supplementary Materials 11, 12).

## Discussion

### Characterization of SSRs in sweet potato

SSRs are useful tools in genetic analysis, germplasm identification, MAS, and tracking of important loci in many crops. SSRs have previously been identified in sweet potato (Buteler et al., 1999; Hu et al., 2004; Schafleitner et al., 2010; Wang et al., 2010; Wang Z. et al., 2011; Tao et al., 2012; Xie et al., 2012), and at least 2099 PCR primers have been designed on the basis of these SSRs since 1999. Of the above-mentioned SSR markers, 142 were derived from the genomic library of sweet potato (Buteler et al., 1999; Hu et al., 2004) and other pairs of primers were designed based on SSR-containing EST sequences. Among these SSR markers, 1214 pairs of PCR primers were found to be polymorphic, or yielded reproducible and strong amplification products when used to analyze different sweet potato accessions (Buteler et al., 1999; Hu et al., 2004; Schafleitner et al., 2010; Wang et al., 2010; Wang Z. et al., 2011). Few studies have examined the usage of these SSR markers in MAS or QTL localization. To collect as many SSR-containing contigs or unigenes as possible, and to design unique SSR primer pairs with high coverage at the genome level, we assembled most of the EST and mRNA sequences available in the public database or derived from high-throughput sequencing *de novo* using an intricate four-step process to obtain the longest unrepeated contigs. We found that sweet potato contains about 63,000 unigenes. This result was also obtained in our recent transcriptome analysis of 17 sweet potato samples (unpublished data), indicating that the sweet potato genome encodes around 63,000 genes.

Furthermore, we examined the characteristics of sweet potato SSRs. We found that most of the identified SSRs are tri- and di-nucleotide repeats, and that the trinucleotide repeat was the most abundant type of SSR. This finding is in agreement with previous research showing that trinucleotide repeat motifs are the most (Wang Z. et al., 2011; Tao et al., 2012) or second most abundant type of SSR in sweet potato, followed by dinucleotide repeats (Schafleitner et al., 2010; Wang et al., 2010; Xie et al., 2012). The four most frequent motif types amongst our identified SSRs were AG/CT (428, 23.465%), AAG/CTT (324, 17.763%), AAT/ATT (150, 8.224%), and AT/TA (126, 6.908%, Figure 3). These motifs were previously shown to occur at the highest frequency in sweet potato EST or cDNA SSRs, especially the AG/CT, AAG/CTT, and AT/TA motifs (Wang et al., 2010; Wang Z. et al., 2011; Tao et al., 2012). AAT/ATT was also reported as an abundant EST SSR motif in sweet potato (Wang et al., 2010; Wang Z. et al., 2011). It is important to note that, the AGA/TCT motif was present at the highest frequency in our identified SSR markers, which were associated with starch content, starch composition, and β-carotene content traits. Thirteen SSR markers identified in this study contained the AGA/TCT motif, including SIP004, SIP29, SIP125, SIP126, SIP127, SIP150, SIP171, SIP183 (Supplementary Material 13), SIP203, SIP226, SIP240, SIP254, and SIP283 (Tables 4–6 and Supplementary Material 2), indicating that this motif should be sought for in future studies of sweet potato SSRs.

### Effects of population structure and estimation model on the association analysis

Li et al. (2008) reported that marker-trait associations vary with the model used for associations with minor effects. In this study, we used four models and various factors to detect marker-trait associations, which took into account trials, experimental years, clone origin, accession type, and kinship. To avoid the influence of undesired population structures that can mimic the signal of associations and lead to false positives or missed real effects (Marchini et al., 2004), we estimated the population structure of 239 sweet potato genotypes using the model-based approach as implemented in STRUCTURE. This method attempts to determine the number and composition of subpopulations based on Hardy-Weinberg equilibrium and linkage equilibrium (Hubisz et al., 2009; Zhang et al., 2014). Our analysis of population structure using 214 SSR primers provided no evidence of significant population structure in the sweet potato collection studied, and the marker-trait associations with the largest effects were consistent across different models, indicating that the population structure (*Q*) has no significant effect on the association analysis in this study. This result was confirmed by model comparison (Supplementary Material 6). However, when the mixed model was used, the number of markers associated with target traits was reduced. As we aimed to select as many informative SSR markers as possible, we collected all the SSR markers identified using various methods and subjected them to further experimental validation. Furthermore, compared with other main crops or model organisms, sweet potato is a genetically challenging hexaploid that can hardly be considered as a model species, and therefore the threshold of significance (*P*-value) used in this study was relatively high.

### Environmental impact on the quality traits of sweet potato

Starch content and composition of storage roots are complex traits controlled by multiple genetic and environmental factors (Li et al., 2008; Rukundo et al., 2013; Schreiber et al., 2014). In this study, we evaluated the quality traits of 239 genotypes over 3 years and used these data for marker-trait association analysis. Among the quality traits measured in the storage root, the dry matter and starch content of each genotype were relatively constant over the 3 experimental years (Table 1), indicating that these parameters were not greatly affected by planting year or weather conditions. Thus, the dry matter and starch content might be mainly controlled by genetic factors and could be regarded as a stable trait in genetic or association analyses. Marker-trait association analysis was based on value measurements taken over 3 years, and most of the selected markers were associated with starch traits in 2 or 3 years. Furthermore, our population included sweet potato genotypes with various dry matter and starch and amylose content characteristics (Figures 1, 2), indicating that this sweet potato panel is suitable for association mapping. Therefore, markers identified as being associated with starch content of the storage root should be reliable.

Several factors may affect the association mapping of β-carotene content and starch composition traits. Firstly, the β-carotene content, amylose content, and A/P in the storage roots of the tested sweet potato genotypes varied markedly over the 3-year study period (Table 1 and Figure 2). A possible reason for this variation is that these traits are easily affected by environmental factors (Laurie et al., 2012; Zhou et al., 2015), such as weather differences. Changes in water conditions may alter the β-carotene content of sweet potato storage roots (Laurie et al., 2012). Therefore, accurate mapping of these traits should take place in multiple locations and over several years. Secondly, the highest β-carotene content measured in our germplasms was almost 8 mg/100 g flesh weight (Tainong 69, measured in 2012), which is less than the highest reported content of 13.83 mg of β-carotene/100 g fresh weight in sweet potato (the genotype ST 14; Nedunchezhiyan et al., 2010). It is possible that the small number of plants with high levels of β-carotene in the flesh of storage root may affect the results of the association analysis. However, slow progress in efforts to breed OFSP lines with high β-carotene content hinders the collection of varieties with a wide range of β-carotene content. In addition, the mechanisms underlying β-carotene formation, accumulation, conversion, and transport in sweet potato storage roots remain unclear, and further studies should focus on the practical applications of these identified markers in breeding practices.

Furthermore, considering that there was no commercial standard sample and mature method for sweet potato amylose measurement, our starch composition-related trait data might be confirmed using novel accurate assessment methods. To enhance the accuracy of marker-trait association, the amylose content and A/P were together used as indicators of starch composition in our association analysis.

### The application potential of SSR markers in MAS of sweet potato

As sweet potato is a highly heterozygous crop, molecular breeding of this crop is a technically challenging goal. As a step toward MAS of sweet potato, we evaluated the ability of these identified markers to detect genotypes with specific trait characteristics. Verifying the ability of these markers to distinguish between cultivars confirmed that the markers had good discriminability and that combinations of these markers could be used to select plants with desirable traits or to predict trait values early in the selection process. Some of the bands differentially amplified in different genotypes were isolated and sequenced. We found that some of the differentiation in the length of the amplified bands resulted from units of SSR motifs, such as those shown in Supplementary Material 13, but some resulted from fragments lacked SSR motifs and had no blast results found in the NCBI-NR database (data not shown).

However, we only detected amplicons of these SSR markers in a small subset of genotypes. Considering the intricate bioprocesses of starch and carotenoid biosynthesis, the complex genetic background of sweet potato and the small proportion of the sweet potato genome that was sampled by SSR markers, it should be established whether all of these SSR markers could be used to identify these traits in all kinds of sweet potato cultivars.

This study represents the first step toward the long-term goal of developing marker-assisted breeding tools that facilitate sweet potato breeding efforts. Furthermore, this research provides two significant resources: (1) A record of direct correlations between marker polymorphisms and trait data that is based on an association analysis performed using a natural population comprised of 239 sweet potato genotypes; (2) A substantial set of SSR markers confirmed to be associated with important quality traits of sweet potato, which can be used to track loci and genome regions or select specific phenotypes in crop breeding programs.

In addition, the SSR markers developed in this study add to the recently accumulated collection of sweet potato molecular markers that can be used for genetic analysis, SSR-based linkage map construction, and further screening for QTLs controlling important traits in sweet potato. We have used the SSR markers identified in this study in QTL mapping of starch-related traits in two sweet potato hybrid populations (unpublished data). Together, these findings enhance our understanding of the mechanisms underlying the inheritance and formation of complex traits in sweet potato, and provide a valuable resource for discovering genes involved in starch and carotenoid biosynthesis in the storage roots of sweet potato.

## Author contributions

KZ wrote the paper, led much of the experimental work, carried out SSR marker development and primer design, analyzed genotypic and phenotypic data, and performed the association analysis. ZW carried out much of the experimental work and performed phenotypic data measurements and screening of SSR markers. DT assisted with field manipulation and natural population handling. CL and YZ helped with field experiments. KL contributed to the statistical analysis. XL and YH helped with field experiments and germplasm management. JW collected germplasms for the study and led the project team.

## Funding

This work was supported by the National Natural Science Foundation of China (31101192), the Application Development Key Project of Chongqing (cstc2013yykfb80010), the Technology Innovation Fund of Chongqing (cstc2015shms-ztzx0121, cstc2015shms-ztzx0128), the Fundamental Research Funds for the Central Universities (XDJK2014B038, XDJK2012C102, 2362015xk05), the National Natural Science Foundation of Chongqing (cstc2012jjB80009, cstc2013jjB80006), and the “111” Project (B12006) of Ministry of Education, P. R. China.

### Conflict of interest statement

The authors declare that the research was conducted in the absence of any commercial or financial relationships that could be construed as a potential conflict of interest.

## Supplementary material

Supplementary Material 1 **Sweet potato germplasm used in this study**.

Supplementary Material 2 **Primer pairs designed based on sweet potato SSRs identified in this study**.

Supplementary Material 3 **Cluster analysis of 239 sweet potato genotypes. Digits represent genotypes corresponding to those in Supplemental Material 1**.

Supplementary Material 4 **STRUCTURE estimation of the number of subpopulations in the entire sweet potato germpalsm collection for *K* ranging from 1 to 20 by likelihood (Ln P(D))**.

Supplementary Material 5 **Two subpopulations inferred from STRUCTURE analysis**. The vertical coordinate indicates the membership coefficients for each individual, and the digits on the horizontal coordinate represent the genotypes corresponding to those shown in Supplemental Material 1. Light gray zone, Pop 1; Dark gray zone, Pop 2.

Supplementary Material 6 **Quantile–quantile plots of estimated -log10 (*P*) from association analysis of four traits using four models (i.e., the naive, *Q, Q*+*K*, and *K* models)**. The black line is the expected line under the null distribution, and the deviations from the expected values indicate that the statistical analysis may cause spurious associations.

Supplementary Material 7 **Marker loci associated with dry matter and starch content of the storage root (*P* < 0.01)**.

Supplementary Material 8 **The amplicons of SSR markers found to be associated with the dry matter and starch content of storage roots shown in Supplementary Material 7**. The first lane of each figure is the molecular weight marker, and the next six lanes are the amplicons obtained using DNA templates from the six accessions (from left to right: D01414, Yushu 33, Xushu 22, S1-5, Chaoshu No. 1, and Shangqiu 52-7, which have average dry matter contents in the storage root of 37.387 ± 0.774, 34.469 ± 2.921, 27.956 ± 1.392, 21.088 ± 1.255, 19.032 ± 0.097, and 13.725 ± 1.803%, respectively, over the 3-year observation period, and average starch contents in the storage root of 26.161 ± 0.673 (>25%), 23.623 ± 2.539 (20–25%), 17.961 ± 1.211 (15–20%), 11.989 ± 1.091 (10–15%), 10.201 ± 0.085 (about 10%), and 5.587 ± 1.568% (< 10%), respectively, over the 3-year period. All samples were genotyped at least in triplicate.

Supplementary Material 9 **Marker loci associated with **β**-carotene content of the storage root (*P* < 0.01)**.

Supplementary Material 10 **Amplicons of SSR markers found to be associated with storage root **β**-carotene content shown in Supplementary Material 9**. The first lane of each figure is the molecular weight marker, and the next six lanes are the amplicons amplified from DNA isolated from the six accessions (from left to right): Ning 4-6, Fengshouhong, Erlangshao, S1-5, Xichengshu 007, and Zhe 147.

Supplementary Material 11 **Marker loci associated with starch composition of the storage root (*P* < 0.01)**. A, amylose content; A/P, amylose/amylopectin content.

Supplementary Material 12 **Amplicons of SSR markers associated with starch composition shown in Supplementary Material 11**. The first lane of each figure is the molecular weight marker, and the next six lanes are amplicons amplified from DNA isolated from the six accessions. Lanes 2–4 (from left to right) are Mianfen No. 1, Nancy Hall, and 0929-106, which had average storage root amylose contents over the 3-year period of 28.465 ± 0.798, 26.081 ± 3.688, and 28.114 ± 1.776%, respectively, and average storage root starch contents over the 3-year period of 30.383 ± 3.713, 19.585 ± 0.231, and 10.857 ± 0.109%, respectively. Lanes 5–7 are Sanheshu, Jishu 52, and Wugonghong, which had average storage root amylose contents over the 3-year period of 15.266 ± 1.656, 13.599 ± 1.206, and 12.076 ± 1.279%, respectively, and average storage root starch contents over the 3-year period of 20.210 ± 2.434, 18.336 ± 1.773, and 11.100 ± 0.824%, respectively.

Supplementary Material 13 **Nucleotide sequences of polymorphic bands generated using a primer pair designed to amplify SSR marker SIP183**.

## References

[B1] Adikini S. Mukasa S. B. Mwanga R. O. M. Gibson R. W. (2015). Sweet potato cultivar degeneration rate under high and low sweet potato virus disease pressure zones in Uganda. Can. J. Plant. Pathol. 37, 136–147. 10.1080/07060661.2015.1004111

[B2] Agili S. Nyende B. Ngamau K. Masinde P. (2012). Selection, yield evaluation, drought tolerance indices of orange-flesh sweet potato (*Ipomoea batatas* Lam) hybrid clone. J. Nutr. Food. Sci. 2, 2–9. 10.4172/2155-9600.1000138

[B3] Anderson M. J. Ter Braak C. J. F. (2003). Permutations tests for multi-factorial analysis of variance. J. Stat. Comput. Simul. 73, 85–113. 10.1080/00949650215733

[B4] Barkley N. A. Roose M. L. Krueger R. R. Federici C. T. (2006). Assessing genetic diversity and population structure in a citrus germplasm collection utilizing simple sequence repeat markers (SSRs). Theor. Appl. Genet. 112, 1519–1531. 10.1007/s00122-006-0255-9 16699791

[B5] Bindler G. Plieske J. Bakaher N. Gunduz I. Ivanov N. Van der Hoeven R.. (2011). A high density genetic map of tobacco (*Nicotiana tabacum* L.) obtained from large scale microsatellite marker development. Theor. Appl. Genet. 123, 219–230. 10.1007/s00122-011-1578-8 21461649 PMC3114088

[B6] Bovell-Benjamin A. C. (2007). Sweet potato: a review of its past, present, and future role in human nutrition. Adv. Food. Nutr. Res. 52, 1–59. 10.1016/S1043-4526(06)52001-7 17425943

[B7] Bradbury P. J. Zhang Z. Kroon D. E. Casstevens T. M. Ramdoss Y. Buckler E. S. (2007). TASSEL: software for association mapping of complex traits in diverse samples. Bioinformatics 23, 2633–2635. 10.1093/bioinformatics/btm308 17586829

[B8] Burri B. J. (2011). Evaluating sweet potato as an intervention food to prevent vitamin A deficiency. Compr. Rev. Food. Sci. Food. Saf. 10, 118–130. 10.1111/j.1541-4337.2010.00146.x

[B9] Buteler M. I. Jarret R. L. LaBonte D. R. (1999). Sequence characterization of microsatellites in diploid and polyploid *Ipomoea*. Theor. Appl. Genet. 99, 123–132. 10.1007/s001220051216

[B10] Cervantes-Flores J. C. Sosinski B. Pecota K. V. Mwanga R. O. M. Catignani G. L. Truong V. D. (2011). Identification of quantitative trait loci for dry-matter, starch, and β-carotene content in sweetpotato. Mol. Breed. 28, 201–216. 10.1007/s11032-010-9474-5

[B11] Chen X. Min D. Yasir T. A. Hu Y. G. (2012). Genetic diversity, population structure and linkage disequilibrium in elite Chinese winter wheat investigated with SSR markers. PLoS ONE 7:e44510. 10.1371/journal.pone.0044510 22957076 PMC3434133

[B12] Collard B. C. Y. MacKill D. J. (2008). Marker-assisted selection: an approach for precision plant breeding in the twenty-first century. Philos. Trans. R. Soc. Lond. B. Biol. Sci. 363, 557–572. 10.1098/rstb.2007.2170 17715053 PMC2610170

[B13] El-Rodeny W. Kimura M. Hirakawa H. Sabah A. Shirasawa K. Sato S.. (2014). Development of EST-SSR markers and construction of a linkage map in faba bean (*Vicia faba*). Breed. Sci. 64, 252–263. 10.1270/jsbbs.64.252 25320560 PMC4154614

[B14] Evanno G. Regnaut S. Goudet J. (2005). Detecting the number of clusters of individuals using the software STRUCTURE: a simulation study. Mol. Ecol. 14, 2611–2620. 10.1111/j.1365-294X.2005.02553.x 15969739

[B15] Firon N. LaBonte D. Villordon A. Kfir Y. Solis J. Lapis E.. (2013). Transcriptional profiling of sweetpotato (*Ipomoea batatas*) roots indicates down-regulation of lignin biosynthesis and up-regulation of starch biosynthesis at an early stage of storage root formation. BMC Genomics 14:460. 10.1186/1471-2164-14-460 23834507 PMC3716973

[B16] Gao F. Gong Y. F. Zhang P. B. (2000). Production and deployment of virus-free sweetpotato in China. Crop. Prot. 19, 105–111. 10.1016/S0261-2194(99)00085-X

[B17] Gibson R. W. Kreuze J. F. (2015). Degeneration in sweetpotato due to viruses, virus-cleaned planting material and reversion: a review. Plant. Pathol. 64, 1–15. 10.1111/ppa.12273

[B18] Gichuru V. Aritua V. Lubega G. Edema R. Adipala E. Rubaihayo P. (2006). A preliminary analysis of diversity among East African sweetpotato landraces using morphological and simple sequence repeats (SSR) markers, in II International Symposium on Sweetpotato and Cassava: Innovative Technologies for Commercialization, eds Tan S. L. (Kuala Lumpur: Acta Hortic, ISHS), 159–164.

[B19] Grabherr M. G. Haas B. J. Yassour M. Levin J. Z. Thompson D. A. Amit I.. (2011). Full-length transcriptome assembly from RNA-Seq data without a reference genome. Nat. Biotechnol. 29, 644–652. 10.1038/nbt.1883 21572440 PMC3571712

[B20] Hamada T. Kim S.-H. Shimada T. (2006). Starch-branching enzyme I gene (*IbSBEI*) from sweet potato (*Ipomoea batatas*); molecular cloning and expression analysis. Biotechnol. Lett. 28, 1255–1261. 10.1007/s10529-006-9083-x 16802100

[B21] Hong J.-H. Kwon Y.-S. Mishra R. K. Kim D. H. (2015). Construction of EST-SSR databases for effective cultivar identification and their applicability to complement for lettuce (*Lactuca sativa* L.) distinctness test. Am. J. Plant. Sci. 6, 113–125. 10.4236/ajps.2015.61013

[B22] Hu J. J. Nakatani M. Lalusin A. G. Kuranouchi T. Fujimura T. (2003). Genetic analysis of sweetpotato and wild relatives usinginter-simple sequence sepeats (ISSRs). Breed. Sci. 53, 297–304. 10.1270/jsbbs.53.297

[B23] Hu J. J. Nakatani M. Mizuno K. Fujimura T. (2004). Development and characterization of microsatellite markers in sweetpotato. Breed. Sci. 54, 177–188. 10.1270/jsbbs.54.177

[B24] Huang J. C. Sun M. (2000). Genetic diversity and relationships of sweetpotato and its wild relatives in *Ipomoea* series *Batatas* (Convolvulaceae) as revealed by inter-simple sequence repeat (ISSR) and restriction analysis of chloroplast DNA. Theor. Appl. Genet. 100, 1050–1060. 10.1007/s001220051386

[B25] Huang L. F. Fang B. P. Chen J. Y. He X. Y. Zhang X. J. Wang Z. Y. (2010). Determination of amylose content in sweet potato by single wavelength colorimetry. J. Chin. Cereals. Oils. Associ. 25, 100–104.

[B26] Hubisz M. J. Falush D. Stephens M. Pritchard J. K. (2009). Inferring weak population structure with the assistance of sample group information. Mol. Ecol. Resour. 9, 1322–1332. 10.1111/j.1755-0998.2009.02591.x 21564903 PMC3518025

[B27] Hwang S. Y. Tseng Y. T. Lo H. F. (2002). Application of simple sequence repeats in determining the genetic relationships of cultivars used in sweet potato polycross breeding in Taiwan. Sci. Hortic. 93, 215–224. 10.1016/S0304-4238(01)00343-0

[B28] Karuri H. W. Ateka E. M. Amata R. Nyende A. B. Muigai A. W. T. (2009). Characterization of Kenyan sweet potato genotypes for SPVD resistance and high dry matter content using simple sequence repeat markers. Afr. J. Biotechnol. 8, 2169–2175.

[B29] Kim S.-H. Hamada T. (2005). Rapid and reliable method of extracting DNA and RNA from sweetpotato, *Ipomoea batatas* (L). Lam. Biotechnol. Lett. 27, 1841–1845. 10.1007/s10529-005-3891-2 16328977

[B30] Kitahara K. Hamasuna K. Nozuma K. Otani M. Hamada T. Shimada T. (2007). Physicochemical properties of amylose-free and high-amylose starches from transgenic sweetpotatoes modified by RNA interference. Carbohydr. Polym. 69, 233–240. 10.1016/j.carbpol.2006.09.025

[B31] Laurie S. M. Faber M. van Jaarsveld P. J. Laurie R. N. du Plooya C. P. Modisane P. C. (2012). β-Carotene yield and productivity of orange-fleshed sweet potato (*Ipomoea batatas* L. Lam.) as influenced by irrigation and fertilizer application treatments. Sci. Hortic. 142, 180–184. 10.1016/j.scienta.2012.05.017

[B32] Li L. Paulo M. J. Strahwald J. Lübeck J. Hofferbert H.-R. Tacke E.. (2008). Natural DNA variation at candidate loci is associated with potato chip color, tuber starch content, yield and starch yield. Theor. Appl. Genet. 116, 1167–1181. 10.1007/s00122-008-0746-y 18379755 PMC2358939

[B33] Li L. Tacke E. Hofferbert H.-R. Lübeck J. Strahwald J. Draffehn A. M.. (2013). Validation of candidate gene markers for marker-assisted selection of potato cultivars with improved tuber quality. Theor. Appl. Genet. 126, 1039–1052. 10.1007/s00122-012-2035-z 23299900 PMC3607734

[B34] Liu K. Muse S. V. (2005). PowerMarker: an integrated analysis environment for genetic marker analysis. Bioinformatics 21, 2128–2129. 10.1093/bioinformatics/bti282 15705655

[B35] Low J. W. Arimond M. Osman N. Cunguara B. Zano F. Tschirley D. (2007). A food-based approach introducing orange-fleshed sweet potatoes increased vitamin A intake and serum retinol concentrations in young children in rural Mozambique. J. Nutr. 137, 1320–1327. 17449599 10.1093/jn/137.5.1320

[B36] Ma D. F. Li Q. Li X. Y. Li H. M. Tang Z. H. Hu L. (2009). Selection of parents for breeding edible varieties of sweetpotato with high carotene content. Agr. Sci. China 8, 1166–1173. 10.1016/S1671-2927(08)60327-2

[B37] Marchini J. Cardon L. R. Phillips M. S. Donnelly P. (2004). The effects of human population structure on large genetic association studies. Nat. Genet. 36, 512–517. 10.1038/ng1337 15052271

[B38] Miah G. Rafii M. Y. Ismail M. R. Puteh A. B. Rahim H. A. Nurul Islam K.. (2013). A review of microsatellite markers and their applications in rice breeding programs to improve blast disease resistance. Int. J. Mol. Sci. 14, 22499–22528. 10.3390/ijms141122499 24240810 PMC3856076

[B39] Mitra S. (2012). Nutritional Status of Orange-Fleshed sweet potatoes in alleviating vitamin A malnutrition through a food-based approach. J. Nutr. Food. Sci. 2, 8–10. 10.4172/2155-9600.1000160

[B40] Myles S. Peiffer J. Brown P. J. Ersoz E. S. Zhang Z. Costich D. E.. (2009). Association mapping: critical considerations shift from genotyping to experimental design. Plant. Cell. 21, 2194–2202. 10.1105/tpc.109.068437 19654263 PMC2751942

[B41] Nedunchezhiyan M. Byju G. Dash S. N. (2010). Effects of organic production of orange fleshed sweet potato (*Ipomoea batatas* L.) on root yield, quality and soil biological health. Int. Res. J. Plant. Sci. 1, 136–143.

[B42] Nedunchezhiyan M. Byju G. Jata S. K. (2012). Sweet potato agronomy. Fruit. Veg. Cereal. Sci. Biotech. 6, 1–10.

[B43] Nei M. (1972). Genetic distance between populations. Am. Nat. 106, 283–292. 20372185

[B44] Rosenberg N. A. (2004). DISTRUCT: a program for the graphical display of population structure. Mol. Ecol. Notes 4, 137–138. 10.1046/j.1471-8286.2003.00566.x

[B45] Roullier C. Rossel G. Tay D. McKey D. Lebot V. (2011). Combining chloroplast and nuclear microsatellites to investigate origin and dispersal of New World sweet potato landraces. Mol. Ecol. 20, 3963–3977. 10.1111/j.1365-294X.2011.05229.x 21880085

[B46] Rukundo P. Shimelis H. Laing M. Gahakwa D. (2013). Storage root formation, dry matter synthesis, accumulation and genetics in sweetpotato. Aust. J. Corp. Sci. 7, 2054–2061.

[B47] Schafleitner R. Tincopa L. Palomino O. Rossel G. Robles R. Alagon R. (2010). A sweetpotato gene index established by *de novo* assembly of pyrosequencing and Sanger sequences and mining for gene-based microsatellite markers. BMC Genomics 11:604 10.1186/1471-2164-11-604 20977749 PMC3017860

[B48] Schmitt A. Rex M. Ebert S. Friedt W. Töpfer R. Zyprian E. (2010). Marker development for important grapevine traits by genetic diversity studies and investigation of differential gene expression, in Methodologies and Results in Grapevine Research, eds Delrot S. Medrano H. Or E. Bavaresco L. Grando S. (London: Springer Science & Business Media), 375–386. 10.1007/978-90-481-9283-0_27

[B49] Schreiber L. Nader-Nieto A. C. Schönhals E. M. Walkemeier B. Gebhardt C. (2014). SNPs in genes functional in starch-sugar interconversion associate with natural variation of tuber starch and sugar content of potato (*Solanum tuberosum* L). G3 4, 1797–1811. 10.1534/g3.114.012377 25081979 PMC4199688

[B50] Tamura K. Dudley J. Nei M. Kumar S. (2007). MEGA4: molecular evolutionary genetics analysis (MEGA) software version 4.0. Mol. Biol. Evol. 24, 1596–1599. 10.1093/molbev/msm092 17488738

[B51] Tao X. Gu Y. H. Jiang Y. S. Zhang Y. Z. Wang H. Y. (2013). Transcriptome analysis to identify putative floral-specific genes and flowering regulatory-related genes of sweet potato. Biosci. Biotechnol. Biochem. 77, 2169–2174. 10.1271/bbb.130218 24200775

[B52] Tao X. Gu Y. H. Wang H. Y. Zheng W. Li X. Zhao C. W.. (2012). Digital gene expression analysis based on integrated *de novo* transcriptome assembly of sweet potato [*Ipomoea batatas* (L.) Lam.]. PLoS ONE 7:e36234. 10.1371/journal.pone.0036234 22558397 PMC3338685

[B53] Teow C. C. Truong V.-D. McFeeters R. F. Thompson R. L. Pecota K. V. Yencho G. C. (2007). Antioxidant activities, phenolic and β-carotene contents of sweet potato genotypes with varying flesh colours. Food. Chem. 103, 829–838. 10.1016/j.foodchem.2006.09.033

[B54] Tumwegamire S. Rubaihayo P. R. LaBonte D. R. Diaz F. Kapinga R. Mwanga R. O. M. (2011). Genetic diversity in white- and orange-fleshed sweetpotato farmer varieties from East Africa evaluated by simple sequence repeat markers. Crop. Sci. 51, 1132–1142. 10.2135/cropsci2010.07.0407

[B55] Varshney R. K. Bertioli D. J. Moretzsohn M. C. Vadez V. Krishnamurthy L. Aruna R.. (2009). The first SSR-based genetic linkage map for cultivated groundnut (Arachis hypogaea L.). Theor. Appl. Genet. 118, 729–739. 10.1007/s00122-008-0933-x 19048225

[B56] Veasey E. A. Borges A. Rosa M. S. Queiroz-Silva J. R. Bressan E. de A. Peroni N. (2008). Genetic diversity in Brazilian sweet potato (*Ipomoea batatas* (L.) Lam., *Solanales, Convolvulaceae*) landraces assessed with microsatellite markers. Genet. Mol. Biol. 31, 725–733. 10.1590/S1415-47572008000400020

[B57] Vimala B. Nambisan B. Hariprakash B. (2011). Retention of carotenoids in orange-fleshed sweet potato during processing. J. Food. Sci. Technol. 48, 520–524. 10.1007/s13197-011-0323-2 23572783 PMC3551189

[B58] Wang M. L. Sukumaran S. Barkley N. A. Chen Z. Chen C. Y. Guo B.. (2011). Population structure and marker-trait association analysis of the US peanut (*Arachis hypogaea* L.) mini-core collection. Theor. Appl. Genet. 123, 1307–1317. 10.1007/s00122-011-1668-7 21822942

[B59] Wang W. Z. Yi F. Du S. R. Wei X. L. Xu L. P. Cao H. L. (1989). Conversion table of the starch content in sweet potato. Acta. Agron. Sin. 15, 94–96.

[B60] Wang Z. Fang B. Chen J. Zhang X. Luo Z. Huang L.. (2010). De novo assembly and characterization of root transcriptome using Illumina paired-end sequencing and development of cSSR markers in sweet potato (*Ipomoea batatas*). BMC Genomics 11:726. 10.1186/1471-2164-11-726 21182800 PMC3016421

[B61] Wang Z. Li J. Luo Z. Huang L. Chen X. Fang B.. (2011). Characterization and development of EST-derived SSR markers in cultivated sweetpotato (*Ipomoea batatas*). BMC Plant Biol. 11:139. 10.1186/1471-2229-11-139 22011271 PMC3206431

[B62] Wickham H. (2009). ggplot2: Elegant Graphics for Data Analysis. New York, NY: Springer-Verlag.

[B63] Xiao Y. Cai D. Yang W. Ye W. Younas M. Wu J.. (2012). Genetic structure and linkage disequilibrium pattern of a rapeseed (*Brassica napus* L.) association mapping panel revealed by microsatellites. Theor. Appl. Genet. 125, 437–447. 10.1007/s00122-012-1843-5 22437490

[B64] Xie F. Burklew C. E. Yang Y. Liu M. Xiao P. Zhang B.. (2012). De novo sequencing and a comprehensive analysis of purple sweet potato (*Impomoea batatas* L.) transcriptome. Planta 236, 101–113. 10.1007/s00425-012-1591-4 22270559

[B65] Yada B. Tukamuhabwa P. Wanjala B. Kim D. J. Skilton R. A. Alajo A. (2010). Characterization of Ugandan sweetpotato germplasm using fluorescent labeled simple sequence repeat markers. HortScience 45, 225–230.

[B66] Yoon M.-Y. Moe K. T. Kim D.-Y. Rho I.-R. Kim S. Kim K.-T. (2012). Genetic diversity and population structure analysis of strawberry (*Fragaria* x *ananassa* Duch.) using SSR markers. Electron. J. Biotechnol. 15, 1–16. 10.2225/vol15-issue2-fulltext-5

[B67] Yu J. M. Pressoir G. Briggs W. H. Vroh Bi I. Yamasaki M. Doebley J. F.. (2006). A unified mixed-model method for association mapping that accounts for multiple levels of relatedness. Nat. Genet. 38, 203–208. 10.1038/ng1702 16380716

[B68] Zhang D. P. Carbajulca D. Ojeda L. Rossel G. Milla S. Herrera C. (2001). Microsatellite Analysis of Genetic Diversity in Sweetpotato Varieties from Latin America. CIP Program Report 1999-2000 (Lima: International Potato Center), 295–301.

[B69] Zhang K. Wu Z. Li Y. Zhang H. Wang L. Zhou Q. (2014). ISSR-based molecular characterization of an elite germplasm collection of sweet potato (*Ipomoea batatas* L.) in China. J. Integr. Agric. 13, 2346–2361. 10.1016/S2095-3119(14)60779-6

[B70] Zhang P. Li J. Li X. Liu X. Zhao X. Lu Y. (2011). Population structure and genetic diversity in a rice core collection (*Oryza sativa* L.) investigated with SSR markers. PLoS ONE 6:e27565. 10.1371/journal.pone.0027565 22164211 PMC3229487

[B71] Zhou W. Yang J. Hong Y. Liu G. Zheng J. Gu Z.. (2015). Impact of amylose content on starch physicochemical properties in transgenic sweet potato. Carbohydr. Polym. 122, 417–427. 10.1016/j.carbpol.2014.11.003 25817686

[B72] Ziska L. H. Runion G. B. Tomecek M. Prior S. A. Torbet H. A. Sicher R. (2009). An evaluation of cassava, sweet potato and field corn as potential carbohydrate sources for bioethanol production in Alabama and Maryland. Biomass Bioenergy 33, 1503–1508. 10.1016/j.biombioe.2009.07.014

