# Supplementary material for: Development and Identification of SSR Markers Associated with Starch Properties and β-Carotene Content in the Storage Root of Sweet Potato (Ipomoea batatas L.)
Source: Front Plant Sci. 2016 Mar 2;7:223. doi: 10.3389/fpls.2016.00223 (PMC4773602; doi:10.3389/fpls.2016.00223)
Supplement: Supplementary Material 1 — Sweet potato germplasm used in this study. [file DataSheet1.pdf]

Supplemental Material 1 Sweet potato germplasm used in this study.

No.	Name	Original source	Storage root flesh color	Pedigree
1	Qinshu No.6	Shanxi, China	yellow	"Hongxin 431" open pollination
2	98-6-23	unknown	pale yellow	genetic material
3	0303-13	unknown	white	breeding line
4	Ningshu192 (D19-2)	Jiangsu, China	orange red	Sushu No.5×Sushu No.4
5	Xushu 25	Jiangsu, China	white	Xushu 18×Xu 781
6	E01-09/Eshu No.9	Hubei, China	pale yellow	868×Zheshu 13
7	Jishu No.2	Shandong, China	light brownish yellow	Huabei 52-45× Guangdong 57-7
8	01160	unknown	white	breeding line
9	Nanfang	Jiangsu, China	white	"Nancy Hall" open pollination
10	Chuanshu 294	Sichuan, China	pale orange red	Jiangjinwujianshao×neiyuan
11	Guangshu 87	Guangdong, China	orange yellow	"Guangshu 69" mass crossing
12	Fushu 7-6	Fujian, China	orange yellow	"Baisheng" mass crossing
13	Baishu No.1	Henan, China	pale yellow	"Anshu 07" self pollination
14	Yushu No.2	Chongqing, China	yellowish white	Nongzhen 868×Wanjinbai
15	Tongrenwutengshao	Guizhou, China	yellow with orange	landrace
16	Mianshu No.4 (88-1070)	Sichuan, China	pale yellow	Xushu 18×Mianfen No.1
17	Sushu No.10 (Ning 15-2)	Jiangsu, China	orange red	Shang 52-7×Sushu No.2
18	Kamoshu	unknown	purple	unknown
19	Jishu 22	Shandong, China	yellow	"Beijing 553" open pollination
20	Jishu No.9	Shandong, China	yellowish white	unknown
21	Chuanshu 34	Sichuan, China	yellowish white	Nanfeng×Xushu 18
22	D0802-1	Sichuan, China	purple	breeding line

23	Fushu 18(Fucaishu 18)	Fujian, China	pale yellow	Quanshu 830×Tainong 71
24	Nongdahong	Beijing, China	pale yellow	Hebei 351×Guangdong natural hybridization
25	Xuzishu No.2 (Xushu 13-4)	Jiangsu, China	purple	Lingzi×Xushu 18
26	Xushu 23 (Xu 87-21-5)	Jiangsu, China	orange red	P616-23× Yanshu 27
27	Fushu 13	Fujian, China	purple	Ayamurasaki× Xiayin No.1
28	2-285	Chongqing, China	white	breeding line
29	Mianshu No.5 (89-1524)	Sichuan, China	white	Zhezhou 84-64×Mianfen No.1
30	Yusu No.8	Chongqing, China	pale yellow	Ning 97-9-2×Nanshu 99
31	Yanshu 20	Shandong, China	yellow	“Yanshu 550”
32	Tainong No.10	Taiwan, China	pale yellow	Meiguohuangpi×Meiguohong
33	Tainong 69	Taiwan, China	orange	mass crossing
34	Manjianshao	unknown	pale yellow	unknown
35	Beniazuma	Japan	pale yellow	introduced variety
36	Zhe 147	Zhejiang, China	white	unknown
37	67-12	Unknown	pale yellow	breeding line
38	Suyu No.1	Jiangsu, China	yellow	Okinawa No.100 ×Nancy Hall
39	Norin 10	Japan	white with purple trace	Jitian×Okinawa No.100
40	Lushu No.10	Shandong, China	pale yellow	unknown
41	Zhezishu No.2	Zhejiang, China	purple	“Zheshu 13” open pollination
42	S1-5	unknown	orange yellow	Germplasm resource
43	Fengshouhuang (65-1596)	Shandong, China	pale yellow	Nancy Hall×Okinawa No.100
44	Nanshu 99	Sichuan, China	pale yellow	Chaoshu 1×Hongpizao
45	Nanshu 88 (81-88)	Sichuan, China	light golden rod yellow	Jinzhuan No.7×Meiguohong
46	Xushu 22	Jiangsu, China	white	Yushu No.7 ×Sushu No.7
47	Yusu 303	Chongqing, China	pale yellow	Ning B58-5×Sushu No.1
48	Jishu 52	Hebei, China	white	unknown

49	Shanchuanzi	Japan	purple	introduced variety
50	Shangqiu 52-7	Henan, China	yellow	Hongqi No.4×19-5
51	Yanshu 5	Fujian, China	orange red	Yanchi red ×Yan 94-1
52	Yanshu 13	Shandong, China	white	Xushu 18 ×Meiguohong
53	Tongzibaipihongxin	Guizhou, China	orange red	landrace
54	Ji 17-1	Hebei, China	pale yellow	wild variety
55	Yanzishu 176 (Yanzishu 2)	Shandong, China	purple	“Zhongzidaozi” open pollination
56	Zheshu 70	Zhejiang, China	orange red	Zheshu 13×Zheshu No.2
57	Yushu 17(6-9-17)	Chongqing, China	orange yellow	Zheshu 13×8129-4
58	Yushu 33	Sichuan, China	cream	“Zheshu 13” mass crossing
59	2-274	Unknown	cream	breeding line
60	Jian 61	Yunnan, China	yellow with purple trace	“Jianshuihuangxin” mass crossing
61	D-1-018	Chongqing, China	yellow	breeding line
62	Yushu Kangbing 1	Guizhou, China	white	“Yushu 868” bud mutation
63	Xiangshao	unknown	orange red	farmer variety
64	Chaoshu No.1	Guangdong, China	yellow	“Qingxinshalaoyue” natural hybridization
65	Jishu 18	Shandong, China	purple	“Xushu 18” open pollination
66	Hongxiaoding	Japan	pale yellow	farmer variety
67	AB940078-1	Chongqing, China	pale yellow	breeding line
68	Sushu No.1 (Nanjing 79-42)	Jiangsu, China	pale red	Nancy Hall×Huabei 52-256
69	Eshu 5 (E 407)	Hubei, China	pale yellow	CN11O8-13×Eshu No.2
70	Sushu 4	Jiangsu, China	orange red	73-51×Bainianshu
71	Zi 807	unknown	pale yellow	germplasm resource
72	Nancy Hall	USA	orange	Farmer variety
73	Zheshu 13	Zhejiang, China	orange yellow	Zhe 3481×Zhe 255
74	Fushu No.8	Fujian, China	Yellow	Fushu No.1×Fushu No.4

75	Fushu No.10	Fujian, China	white	Fushu 7-6×Tainong 71
76	Beijing 553 (50-553)	Beijing, China	apricot yellow	“Okinawa No.100” open pollination
77	0415-2	unknown	white	breeding line
78	Chuanshu 217 (chuan 12-17)	Sichuan, China<br>Chongqing, China	white	Jishu 98×Liyuan No.1
79	Xushu 18 (73-2518)	Jiangsu, China	white	Xindazi×Huabei 52-45
80	Mianfen No.1 (82-1564)	Sichuan, China	yellowish white	79-14×79—96
81	D01414	Chongqing, China	Pale yellow	germplasm resource
82	Z0838-6	unknown	pale yellow	breeding line
83	0423-3	unknown	pale yellow	breeding line
84	9415-45	unknown	pale yellow	breeding line
85	Mian E01-19	Sichuan, China	purple	germplasm resource
86	Virus-Free 4553	Chonqing, China	pale yellow	virus-free material
87	Rizi No.5	Japan	purple	introduced variety
88	0146-30	Chongqing, China	white	Nanfeng×Chaoshu No.1
89	Wugonghong	Shanxi, China	pale yellow	unknown
90	Mianzishu No.9	Sichuan, China	purple	“4-4-259” mass crossing
91	Jishu 15	Shandong, China	pale yellow	Ji 85003×Ji 78268
92	Yushuwang (YSW-2)	Chongqing, China	white	Nongzhen 868×Wanjinbai
93	Wan 9610-6	Chongqing, China	white	breeding line
94	00130-283	unknown	white	breeding line
95	Rizi No.9	Japan	purple	introduced variety
96	8715-794	unknown	white	breeding line
97	QZ0570	Zhejiang, China	purple	“Zhecaishu726” open pollination
98	D218-4 (0218-4)	Chongqing, China	orange red	“Mianfen No.1” mass crossing
99	4-3-218	Chongqing, China	pale yellow	Sushu No.2×Zhe 13

100	Ning 29-11	Jiangsu, China	purple	Xushu 18 ×Zhezishu No.1
101	962046	unknown	pale yellow	breeding line
102	Yushu 6 (0506-406)	Chongqing, China	pale yellow	“Zheshu 13” mass crossing
103	Wanzi 11	Chongqing, China	purple	“Xushu 22” mass crossing
104	Wan 9601-131	Chongqing, China	orange yellow	breeding line
105	011-45	unknown	white	unknown
106	Wengcaizhong	Guangdong, China	yellowish white	farmer variety
107	Xinxiang	Zhejiang, China	yellow	Jinyu ×Zheshu No.2
108	Yuzi 263	Chongqing, China	purple	“Xushu 18” mass crossing
109	Xichengshu 007 (Nanshu 007)	Sichuan, China	yellow	BB18-152×9014-3
110	Yushu 4 (0610-54)	Chongqing, China	pale yellow with purple trace	“Zheshu 13” mass crossing
111	Sanheshu	Zhejiang, China	white	88-3×Nanjing 118
112	Wanshu No.5 (0515-5)	Chongqing, China	white	Xu 55-2× 92-3-7
113	Fenghuang	unknown	pale yellow	germplasm resource
114	Wanshu 562	Chongqing, China	purple	“Rizi No.13” mass crossing
115	Wanzi No.5	Chongqing, China	purple	“Wanzishu 56” mass crossing
116	Diaosihong	USA	yellow with a tinge of orange	introduced variety
117	9501-6	Unknown	pale yellow	breeding line
118	Norin 1	Japan	pale yellow	Yuanqi×Qifu
119	0619-7	Chongqing, China	orange red	breeding line
120	Nanzishu 008	Sichuan, China	purple	“Ribenzishu” mass crossing
121	0404-4	unknown	orange red	breeding line
122	Mianshuzaoqiu (Zaoziu No.64)	Sichuan, China	pale yellow	79-75×Zaoshuhong
123	Zhengan Red-skined	Guizhou, China	white	landrace
124	Rizi No.7	Japan	purple	introduced variety
125	0508-2	Chongqing, China	white	breeding line

126	Zhezishu No.1	Zhejiang, China	purple	Ningzishu No.1×Zheshu 13
127	Zheshu 23	Zhejiang, China	orange yellow	Zheshu 13×Zheshu 132
128	Shangshu 19 (968-19)	Henan, China	white	SL-01×Yushu No.7
129	Chuancaishu 211	Sichuan, China	white	"Guangshucai No.2" open pollination
130	Jiangjinwujianshao	Chongqing, China	orange yellow	landrace
131	Wanzi 51	Chongqing, China	purple	"Ribenzishu" mass crossing
132	Wan 9319-1	Chongqing, China	white	breeding line
133	Ji 78268	Shandong, China	yellow	unknown
134	Ya 4	Chongqing, China	pale yellow	unknown
135	Chuanshu 124	Sichuan, China	orange yellow	Chuanshu 101×8816-60
136	Xichong huangxin	Sichuan, China	orange yellow	farmer variety
137	Wanshu 90	Chongqing, China	yellow	unknown
138	Ning 97-5	Jiangsu, China	pale yellow	unknown
139	Hongpizao	Sichuan, China	pale yellow	Huabei 117×Wushan Hunan shao
140	Neijiang No.3	Sichuan, China	white	landrace
141	Luoxushu No.9	Henan, China<br>Jiangsu, China	pale yellow	Xu 781×Xushu 18
142	Jiagoudazi	unknown	pale yellow	germplasm resource
143	9809-7	unknown	white	breeding line
144	Wanzi 53	Chongqing, China	purple	unknown
145	0409-17	Chongqing, China	yellow with purple trace	"Mianfen No.1" mass crossing
146	Guizhou Hongshao	Guizhou, China	yellow	landrace
147	Wanshu 34	Chongqing, China	yellow	87-1227×8410-788
148	5-12-17	Chongqing, China	white	Jishu 98×Liyuan No.1
149	0317-6	Chongqing, China	pale yellow	Xu 55-2×Mianfen No.1
150	Ruishu No.1	Zhejiang, China	pale yellow	Pengwei×Meijianhong

151	92-93	unknown	orange	breeding line
152	Wanhua No.7	Chongqing, China	pale yellow	unknown
153	Nanjing 92	Jiangsu, China	pale yellow	Jiagoudazi×52-45
154	Xiangnonghuangpi	Hunan, China	orange red	Okinawa No.100×Nancy Hall
155	Virus-Free No.10	Chongqing, China	orange red	virus-Free material
156	Jewel	USA	orange	introduced variety
157	9413-7	unknown	white	breeding line
158	Wanzi 33	Chongqing, China	purple	unknown
159	Chengdu Red	Sichuan, China	yellow	landrace
160	Rizi No.4	Japan	purple	introduced variety
161	Beijing Red	Hebei, China	pale yellow	unknown
162	Virus-Free No.273	Chongqing, China	purple	virus-free material
163	Wanzishu 56 (Wanzi 56)	Chongqing, China	purple	"Ribenzixin" mass crossing
164	Jishu 98 (Ji 17-4)	Hebei, China	pale yellow	Ji 21-2×Y-6
165	Yushu 14	Henan, China	pale yellow	unknown
166	Sushu 3 (83-1-289)	Jiangsu, China	white	Xushu 18×Qunli No.2
167	Wan 9811-1-9	Chongqing, China	pale yellow	breeding line
168	Zidawo	Unknown	purple	genetic material
169	Nanchong 5-155	Sichuan, China	pale orange yellow	genetic material
170	Ri 19	Japan	yellow with orange	"Ribenzishu" Mass crossing
171	Dianjiangdabaishao	Chongqing, China	pale yellow	landrace
172	Feng 7-105	Jiangsu, China	white	unknown
173	Niriliya	Nigeria	white	introduced variety
174	Yanshu No.5	Shandong, China	orange yellow	unknown
175	Liushiri (Yubeibai)	Fujian, China	white	farmer variety
176	Xu 781	Jiangsu, China	pale yellow	selected from breeding lines introduced from

		International Potato Center (CIP)		CIP
177	Xu 17-10	Jiangsu，China	pale yellow	unknown
178	05ZZ-1	Chongqing, China	white	“8410-788” mass crossing
179	0601-3	Chongqing, China	white	breeding line
180	D01593	Sichuan, China	white	“D-3-037” mass crossing
181	Wanchun No.1	Chongqing, China	orange red	unknown
182	Ernanshao	unknown	orange red	farmer variety
183	Fengshouhong	unknown	orange red	unknown
184	Nanshu 95	Sichuan, China	yellow	Xushu 18×Wanchun No.1
185	91-7340	Sichuan, China	white	breeding line
186	Chuanshu 69	Sichuan, China	pale yellow	788×Chuanshu 34
187	0421-4	Chongqing, China	white	breeding line
188	Ningshu 180 (Ning 180)	Jiangsu，China	white	unknown
189	Guangshu 95-145	Guangdong, China	orange yellow	Guangshu 128×Guangshu 88-70
190	8410-788	Sichuan, China	white	Nanfeng×Xushu 18
191	Fu 7-16	Anhui, China	white	unknown
192	Chuan 618-25	Sichuan, China	white	Biaoxinhong×Xiaobaiteng
193	Wan 9601-132	Wanzhou, China	white	breeding line
194	Xiaoxian Xiaohuaye	Anhui, China	cream	farmer vareity
195	Virus-Free No.5	Chongqing, China	pale yellow	virus-free material
196	4-4-259	unknown	yellow	breeding line
197	Fulingdabaishao	Sichuan, China	white	land race
198	Mianshu No.6	Sichuan, China	white, or with purple trace	Xushu 18×83-1229
199	Xiangshu No.6 (74-121)	Hunan, China	pale yellow	Huabei 48×Lizixiang
200	Xushu 29-2	Jiangsu, China	white	unknown

201	Ning 23-1	Jiangsu, China	yellow	“Suyu 303” open pollination
202	Yongshu 14	unknown	yellow	unknown
203	Yucai No.1	Chongqing, China	orange red	unknown
204	9824-1-4	unknown	pale orange yellow	unknown
205	Beijing No.2	Beijing, China	yellow with orange	Pengwei×Zaoshuhong
206	Yushu No.7	Henan, China	white	Nanfeng×Xushu 18 line 78-28
207	Yushu No.8	Henan, China	white, some have purple trace	Pengwei ×Xiaobaiteng
208	Dananfu	Fujian, China	orange red	Nancy Hall×Wuguqilong
209	Satsumahikari	Japan	pale yellow	introduced variety
210	Wanshu No.4	Anhui, China	white with purple trace	Fushu No.2×Norin 11
211	Ningzishu 1	Jiangsu, China	purple	“Ning 97-23” open pollination
212	0912-5	unknown	white	Breeding line
213	Zheshu 132	Zhejiang, China	orange red	Zheshu 13×Zheshu 3481
214	200850	unknown	yellow with orange	breeding line
215	0929-106	unknown	yellow	breeding line
216	E0611-2	unknown	purple	breeding line
217	Nongfu No.8	unknown	white	unknown
218	Wanshu No.7	Chongqing, China	orange red	“Fenghuang” mass crossing
219	0315-8	unknown	white	breeding line
220	H11-36	Jiangsu, China	white	breeding line
221	Enshu No.2	Hubei, China	orange red	8714-13×Xushu 18
222	Luoxushu No.8	Henan, China	white	Xushu 18×Xu 781
223	Kokei 14	Japan	white with pale yellow	introduced variety
224	96403-141	unknown	pale yellow	breeding line
225	Qinshu No.7	Shanxi, China	orange red	Qinshu No.4× Hongxin 431
226	Fuxushu No.6 (Fuxu 213-6)	Anhui, China	pale yellow	“Lizixiang” open pollination

		Jiangsu, China		
227	Tongrengedoushao	Guizhou, China	orange yellow	landrace
228	Mianshu No.7 (92-1229)	Sichuan, China	pale yellow	Xushu 18×8410-788
229	Jishu 99	Hebei, China	pale yellow	Ji 21-2×Y-6
230	Wan 892-45	Chongqing, China	white	breeding line
231	7-S	unknown	orange red	unknown
232	0621-1	unknown	white	breeding line
233	Kyushu 55	Japan	white	introduced variety
234	Wengan Yellow-fleshed	Guizhou, China	yellow	landrace
235	lizixiang	Jiangsu, China	yellow	Nancy hall ×Okinawa No.100
236	Zheshu 6025	Zhejiang, China	yellow	Zheshu 27-5×Zheshu No.2
237	1028-1 Mottled leaf	Chongqing, China	orange yellow	Mutation
238	Sushu No.8	Jiangsu, China	apricot yellow	Sushu No.4×Sushu No.1
239	Okinawa No. 100	Japan	pale yellow	Qifu ×Chaozhou
