# Supplementary figures and images for: Development and Identification of SSR Markers Associated with Starch Properties and β-Carotene Content in the Storage Root of Sweet Potato (Ipomoea batatas L.)
Source: Front Plant Sci. 2016 Mar 2;7:223. doi: 10.3389/fpls.2016.00223 (PMC4773602; doi:10.3389/fpls.2016.00223)

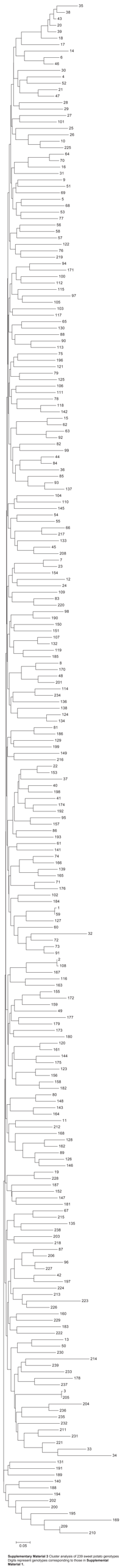

Supplement: Supplementary Material 3 — Cluster analysis of 239 sweet potato genotypes. Digits represent genotypes corresponding to those in Supplemental Material 1. [file DataSheet3.PDF]
