# Supplementary material for: Development and Identification of SSR Markers Associated with Starch Properties and β-Carotene Content in the Storage Root of Sweet Potato (Ipomoea batatas L.)
Source: Front Plant Sci. 2016 Mar 2;7:223. doi: 10.3389/fpls.2016.00223 (PMC4773602; doi:10.3389/fpls.2016.00223)
Supplement: Supplementary Material 4 — STRUCTURE estimation of the number of subpopulations in the entire sweet potato germpalsm collection for K ranging from 1 to 20 by likelihood (Ln P(D)). [file DataSheet4.pdf]

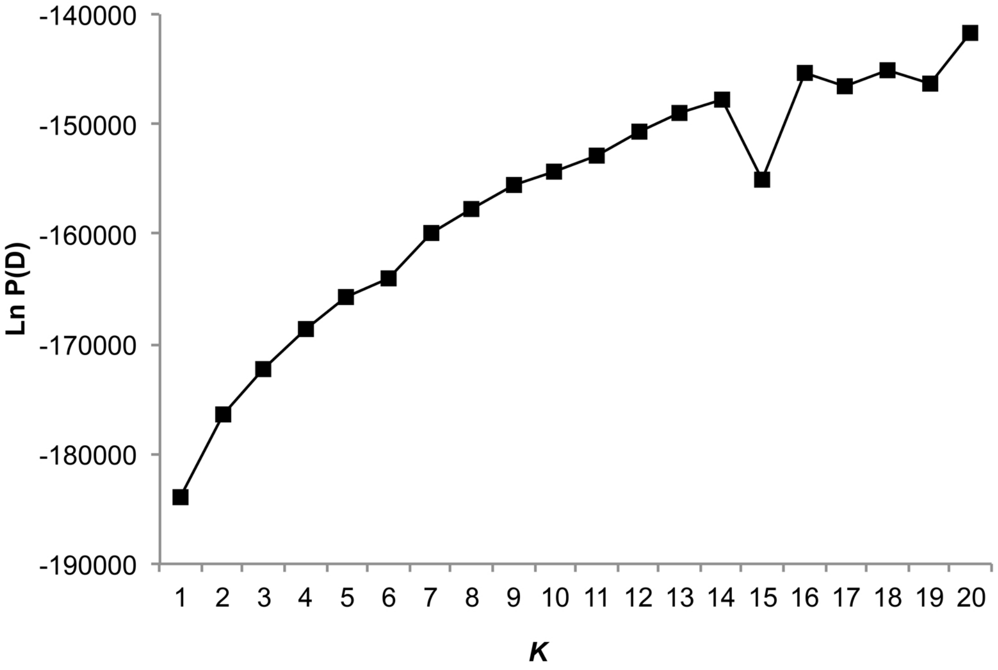

Supplementary Material 4 STRUCTURE estimation of the number of subpopulations in the entire sweet potato germpalsm collection for *K* ranging from 1 to 20 by likelihood (Ln P(D))
