# Supplementary material for: Development and Identification of SSR Markers Associated with Starch Properties and β-Carotene Content in the Storage Root of Sweet Potato (Ipomoea batatas L.)
Source: Front Plant Sci. 2016 Mar 2;7:223. doi: 10.3389/fpls.2016.00223 (PMC4773602; doi:10.3389/fpls.2016.00223)
Supplement: Supplementary Material 5 — Two subpopulations inferred from STRUCTURE analysis. The vertical coordinate indicates the membership coefficients for each individual, and the digits on the horizontal coordinate represent the genotypes corresponding to those shown in Supplemental Material 1. Light gray zone, Pop 1; Dark gray zone, Pop 2. [file DataSheet5.pdf]

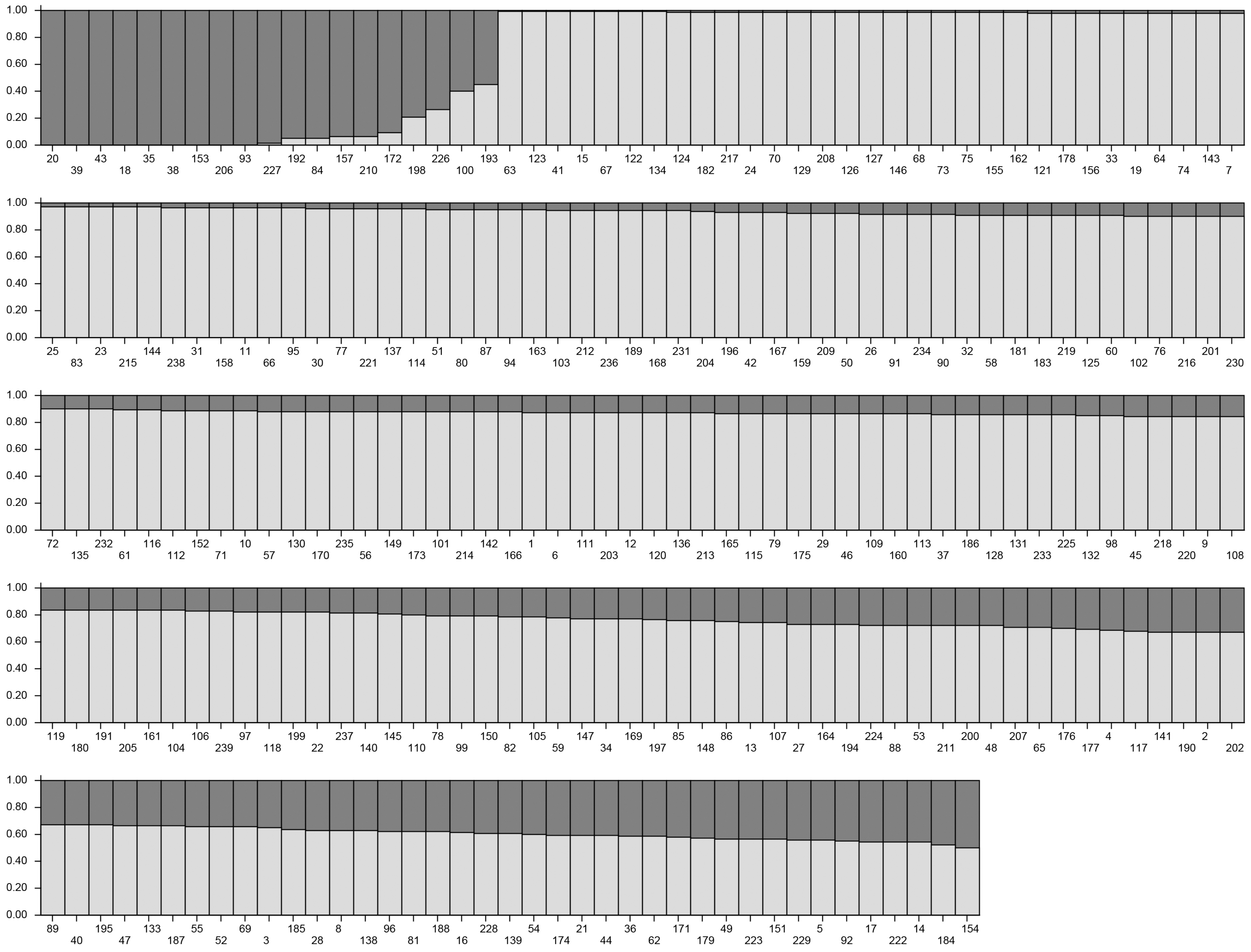

Supplementary Material 5 Two subpopulations inferred from STRUCTURE analysis

The vertical coordinate indicates the membership coefficients for each individual, and the digits on the horizontal coordinate represent the genotypes corresponding to those shown in Supplemental Material 1. Light gray zone, Pop 1; Dark gray zone, Pop 2.
