# Supplementary material for: Development and Identification of SSR Markers Associated with Starch Properties and β-Carotene Content in the Storage Root of Sweet Potato (Ipomoea batatas L.)
Source: Front Plant Sci. 2016 Mar 2;7:223. doi: 10.3389/fpls.2016.00223 (PMC4773602; doi:10.3389/fpls.2016.00223)
Supplement: Supplementary Material 6 — Quantile–quantile plots of estimated -log10 (P) from association analysis of four traits using four models (i.e., the naive, Q, Q+K, and K models). The black line is the expected line under the null distribution, and the deviations from the expected values indicate that the statistical analysis may cause spurious associations. [file DataSheet6.pdf]

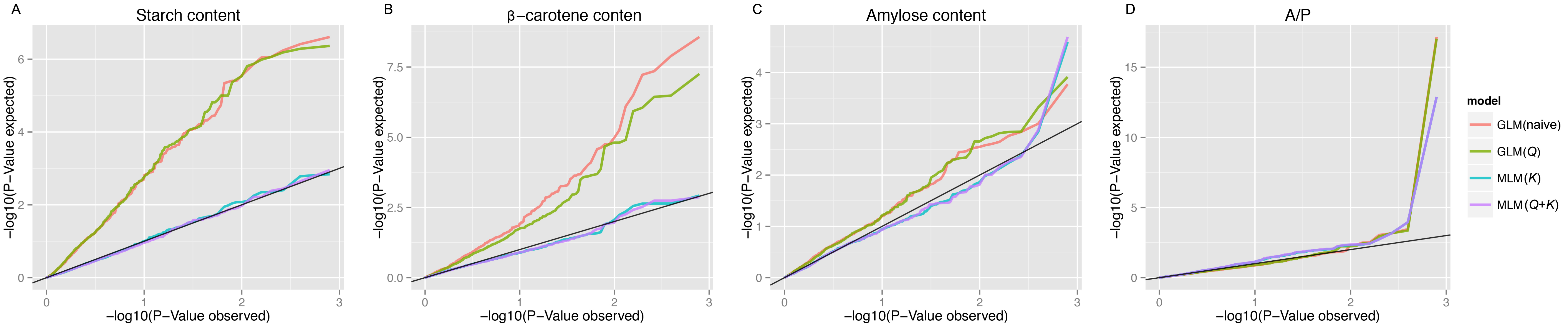

Supplementary Material 6 Quantile–quantile plots of estimated -log10 (*P*) from association analysis of four traits using four models (i.e., the naive, *Q*, *Q+K* and *K* models)
The black line is the expected line under the null distribution, and the deviations from the expected values indicate that the statistical analysis may cause spurious associations.
