# Supplementary material for: Development and Identification of SSR Markers Associated with Starch Properties and β-Carotene Content in the Storage Root of Sweet Potato (Ipomoea batatas L.)
Source: Front Plant Sci. 2016 Mar 2;7:223. doi: 10.3389/fpls.2016.00223 (PMC4773602; doi:10.3389/fpls.2016.00223)
Supplement: Supplementary Material 7 — Marker loci associated with dry matter and starch content of the storage root (P < 0.01). [file DataSheet7.pdf]

Supplementary Material 7 Marker loci associated with dry matter and starch content of the storage root (*P*<0.01).

		Based on phenotypic data Y2011		Based on phenotypic data Y2012		Based on phenotypic data Y2013		Based on phenotypic data 3 years	
Marker	Model used	*P* value	R^2	*P* value	R^2	*P* value	R^2	*P* value	R^2
SIP019	1, 2							8.27E-04	0.05547
				0.00855	0.08988			0.00165	0.04934
		9.20E-04	0.11669			0.0031	0.21299	2.28E-06	0.11177
SIP026	1, 2							0.00106	0.05528
								0.00207	0.04909
SIP037	1–4	1.23E-05	0.19422	2.05E-04	0.20989			1.25E-09	0.17775
						0.00404	0.19956		
SIP039	1, 2							7.77E-05	0.06453
		0.00652	0.08023	0.00144	0.12835			1.55E-05	0.07657
SIP054	1							0.00143	0.05252
								0.00189	0.04994
SIP068	1							0.00432	0.04229
SIP090	1–4					0.00891	0.16858	2.42E-04	0.056
								0.0028	0.03757
SIP091	1, 2							5.44E-04	0.04991
SIP106	1, 2	1.34E-04	0.15198	0.00181	0.15327			1.46E-07	0.13638
		2.82E-04	0.13845	0.00867	0.11108			3.98E-06	0.10672
SIP114	1, 2							0.00221	0.03936
SIP118	1, 2							0.00734	0.03038
SIP139	3, 4					0.0091	0.05258		
SIP142	1, 2			0.00327	0.04082			2.93E-04	0.03137
								0.00112	0.02546
SIP150	1–4	5.05E-04	0.15767					0.00139	0.02417
SIP154	1–4			2.01E-04	0.06416			7.36E-05	0.03721
				8.29E-04	0.05218			8.92E-05	0.03636
SIP161	1–4							1.55E-04	0.03347
		0.00311	0.10974					2.66E-04	0.03135
SIP172	1, 2	2.57E-04	0.16006						
		2.57E-04	0.16006						
SIP193	1, 2	0.00833	0.09277					0.00386	0.01966
SIP205	1–4	0.00997	0.08674	0.00813	0.0331			2.27E-04	0.03218
		0.00689	0.09492					2.58E-04	0.03161
SIP207	1, 2					0.00884	0.05098	7.50E-04	0.02702
								7.11E-04	0.02725

SIP211	1, 2			0.00826	0.0325			2.11E-04	0.03194
SIP216	1–4	0.00757	0.09409					0.0027	0.02164
SIP218	1, 2	0.00206	0.1171			9.43E-04	0.0801	5.99E-04	0.03332
		0.00552	0.09619	0.0049	0.03715			0.00495	0.02247
SIP228	1, 2			0.00995	0.03151			0.00136	0.02468
								0.00276	0.0216
SIP229	1, 2	0.00764	0.09059						
		0.00764	0.09059						
SIP233	1–4					6.45E-04	0.08704	0.00264	0.02196
SIP234	1–4	1.29E-04	0.19016			0.00305	0.06503	1.03E-06	0.05601
								1.64E-04	0.03373
SIP243	1–4					0.00755	0.05224	0.00134	0.029
						0.00311	0.06362	1.94E-04	0.03897
								0.00495	0.01863
SIP246	1–4							0.00895	0.01614
				0.00899	0.03195			2.49E-04	0.03145
		5.02E-04	0.14881					1.15E-04	0.03498
		2.91E-05	0.20689					4.68E-05	0.03888
SIP262	1, 2	2.67E-04	0.162					2.29E-04	0.03197
		0.0012	0.1304					2.10E-04	0.03234
		0.0018	0.1217					7.55E-04	0.02679
SIP279	1, 2	0.00121	0.13202					0.00814	0.01682
SIP292	1, 2	0.00559	0.10319	0.00513	0.03687			4.01E-05	0.03978
SIP299	1, 2			0.00396	0.03873			0.00204	0.02241
SIP302	1–4							0.00354	0.02406
								0.00482	0.0225
