# Supplementary material for: Development and Identification of SSR Markers Associated with Starch Properties and β-Carotene Content in the Storage Root of Sweet Potato (Ipomoea batatas L.)
Source: Front Plant Sci. 2016 Mar 2;7:223. doi: 10.3389/fpls.2016.00223 (PMC4773602; doi:10.3389/fpls.2016.00223)
Supplement: Supplementary Material 8 — The amplicons of SSR markers found to be associated with the dry matter and starch content of storage roots shown in Supplementary Material 7. The first lane of each figure is the molecular weight marker, and the next six lanes are the amplicons obtained using DNA templates from the six accessions (from left to right: D01414, Yushu 33, Xushu 22, S1-5, Chaoshu No. 1, and Shangqiu 52-7, which have average dry matter contents in the storage root of 37.387 ± 0.774, 34.469 ± 2.921, 27.956 ± 1.392, 21.088 ± 1.255, 19.032 ± 0.097, and 13.725 ± 1.803%, respectively, over the 3-year observation period, and average starch contents in the storage root of 26.161 ± 0.673 (>25%), 23.623 ± 2.539 (20–25%), 17.961 ± 1.211 (15–20%), 11.989 ± 1.091 (10–15%), 10.201 ± 0.085 (about 10%), and 5.587 ± 1.568% (< 10%), respectively, over the 3-year period. All samples were genotyped at least in triplicate. [file DataSheet8.pdf]

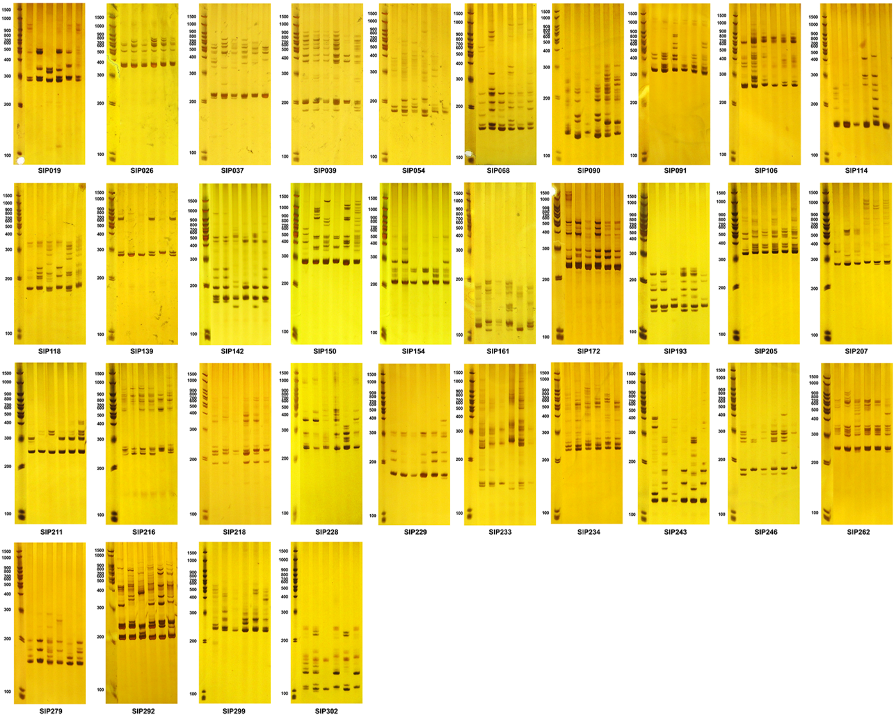

Supplementary Material 8 The amplicons of SSR markers found to be associated with the dry matter and starch content of storage roots shown in **Supplementary Material 7**

The first lane of each figure is the molecular weight marker, and the next six lanes are the amplicons obtained using DNA templates from the six accessions (from left to right: D01414, Yushu 33, Xushu 22, S1-5, Chaoshu No.1, and Shangqiu 52-7, which have average dry matter contents in the storage root of 37.387±0.774, 34.469±2.921, 27.956±1.392, 21.088 ±1.255, 19.032±0.097, and 13.725±1.803%, respectively, over the 3-year observation period, and average starch contents in the storage root of 26.161±0.673 (>25%), 23.623±2.539 (20-25%), 17.961±1.211 (15-20%), 11.989±1.091 (10-15%) , 10.201±0.085 (about 10%), and 5.587±1.568% (<10%), respectively, over the 3-year period. All samples were genotyped at least in triplicate.
