# Supplementary material for: Development and Identification of SSR Markers Associated with Starch Properties and β-Carotene Content in the Storage Root of Sweet Potato (Ipomoea batatas L.)
Source: Front Plant Sci. 2016 Mar 2;7:223. doi: 10.3389/fpls.2016.00223 (PMC4773602; doi:10.3389/fpls.2016.00223)
Supplement: Supplementary Material 9 — Marker loci associated with β-carotene content of the storage root (P < 0.01). [file DataSheet9.pdf]

Supplementary Material 9 Marker loci associated with β-carotene content of the storage root (P<0.01).

Marker	Model used	Based on phenotypic data Y2011		Based on phenotypic data Y2012		Based on phenotypic data Y2013		Based on phenotypic data 3 years	
		P value	R^2	*P* value	R^2	*P* value	R^2	*P* value	R^2
SIP001	1–4			1.27E-05	0.68371			0.00838	0.07315
SIP028	1–4					0.00683	0.1862	0.00469	0.05988
SIP031	1, 2			0.00138	0.46132			0.00601	0.05662
SIP060	1–4	0.0058	0.08671	9.15E-04	0.48562			0.00993	0.07008
SIP068	1, 2					0.00184	0.23898	0.00572	0.05725
						0.00184	0.23898	0.00296	0.06591
SIP098	1–4					7.49E-04	0.2738		
						0.00101	0.26257		
SIP140	1, 2					4.23E-04	0.10118	1.91E-04	0.07583
						9.93E-04	0.08813	8.26E-04	0.06139
SIP142	1, 2			0.00217	0.15329			0.00713	0.04065
				8.08E-04	0.18011			0.00749	0.04015
SIP148	1, 2					0.00283	0.0743	8.55E-04	0.06207
SIP157	3, 4					0.00805	0.06142		
SIP166	1–4			0.00238	0.1584			0.00322	0.03807
				7.19E-04	0.19247			2.07E-04	0.05975
SIP172	1, 2			0.00253	0.14904			4.66E-05	0.06828
				1.07E-04	0.23316			6.29E-04	0.04862
SIP183	1, 2							0.00131	0.0429
								0.00395	0.04597
SIP193	1							0.00131	0.05681
								4.39E-04	0.06762
SIP197	1–4			0.00208	0.15436	4.20E-05	0.13307	3.21E-07	0.10457

								0.00179	0.04039
SIP204	1, 2			0.00764	0.11832				
SIP209	3, 4					0.00456	0.0711		
SIP215	1, 2					0.00552	0.06342	0.00577	0.03184
SIP216	1–4					5.10E-05	0.1314	4.28E-07	0.13627
SIP232	3, 4	0.00866	0.12594						
				6.95E-05	0.24803			1.29E-08	0.12885
				4.11E-04	0.20133			5.58E-06	0.0842
SIP262	1–4			5.40E-05	0.25445			7.95E-07	0.0987
				6.95E-05	0.24803			5.98E-08	0.11772
				0.00451	0.13524	0.00226	0.07629	2.64E-05	0.07254
SIP264	1, 2							0.00568	0.03182
SIP266	1, 2			7.11E-04	0.18654				
SIP275	1, 2							0.00627	0.04215
SIP286	1, 2			0.00994	0.12106				
SIP295	1, 2							5.49E-04	0.05007
SIP301	1, 2			0.00177	0.1614			0.00411	0.03464
SIP306	1–4	1.10E-05	0.28552						
