# Supplementary material for: Development and Identification of SSR Markers Associated with Starch Properties and β-Carotene Content in the Storage Root of Sweet Potato (Ipomoea batatas L.)
Source: Front Plant Sci. 2016 Mar 2;7:223. doi: 10.3389/fpls.2016.00223 (PMC4773602; doi:10.3389/fpls.2016.00223)
Supplement: Supplementary Material 10 — Amplicons of SSR markers found to be associated with storage root β-carotene content shown in Supplementary Material 9. The first lane of each figure is the molecular weight marker, and the next six lanes are the amplicons amplified from DNA isolated from the six accessions (from left to right): Ning 4-6, Fengshouhong, Erlangshao, S1-5, Xichengshu 007, and Zhe 147. [file DataSheet10.PDF]

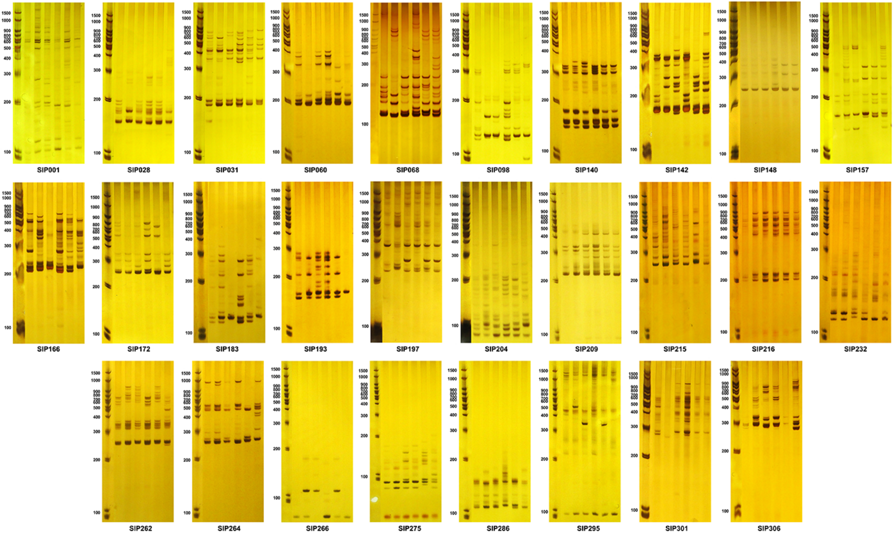

Supplementary Material 10 Amplicons of SSR markers found to be associated with storage root β-carotene content shown in **Supplementary Material 9**

The first lane of each figure is the molecular weight marker, and the next six lanes are the amplicons amplified from DNA isolated from the six accessions (from left to right): Ning 4-6, Fengshouhong, Erlangshao, S1-5, Xichengshu 007, and Zhe 147.
