# Supplementary material for: Development and Identification of SSR Markers Associated with Starch Properties and β-Carotene Content in the Storage Root of Sweet Potato (Ipomoea batatas L.)
Source: Front Plant Sci. 2016 Mar 2;7:223. doi: 10.3389/fpls.2016.00223 (PMC4773602; doi:10.3389/fpls.2016.00223)
Supplement: Supplementary Material 11 — Marker loci associated with starch composition of the storage root (P < 0.01). A, amylose content; A/P, amylose/amylopectin content. [file DataSheet11.pdf]

Supplementary Material 11 Marker loci associated with starch composition of the storage root (*P*<0.01). A, amylose content; A/P, amylose/ amylopectin content.

Marker	Model used	Based on phenotypic data Y2011			Based on phenotypic data Y2012			Based on phenotypic data Y2013			Based on phenotypic data over 3 years		
		Trait	*P* value	R^2	Trait	*P* value	R^2	Trait	*P* value	R^2	Trait	*P* value	R^2
SIP019	1–4										A	0.00848	0.04779
											A	0.00869	0.0475
											A	0.00659	0.05083
		11A	0.007	0.08958							A	0.00579	0.05238
		11A/P	0.00447	0.09902									
SIP021	1							13 A/P	0.00321	0.21164	A/P	0.00545	0.04188
SIP027	1–4				12A	0.0077	0.09695				A	0.00463	0.04195
					12 A/P	4.26E-04	0.1777				A/P	0.0022	0.06047
SIP028	1	11A	0.00837	0.08581							A	0.00479	0.05468
								13 A/P	0.00607	0.18631			
SIP037	3, 4				12A	0.00405	0.14145				A	7.59E-04	0.08282
SIP039	3, 4										A	0.00257	0.05124
											A/P	0.00964	0.04815
SIP042	3, 4	11A	0.00615	0.09758				13 A/P	0.00107	0.33145			
SIP054	1							13A	0.00737	0.17851			
SIP090	3, 4										A/P	0.00896	0.04912
SIP091	1–4				12A	0.00205	0.1383	13A	0.00966	0.1684	A	3.65E-04	0.06624
								13 A/P	0.00973	0.16097			
SIP098	1	11A	0.00491	0.09703							A	0.00349	0.0585
SIP101	1–4										A	0.00502	0.05412
								13 A/P	0.00233	0.22417	A/P	0.00408	0.05341

					12A	0.00469	0.12188				A	0.00521	0.05368
											A	9.96E-04	0.05826
		11A	0.0053	0.09542							A	4.61E-04	0.08304
SIP106	1												
		11A	0.00243	0.11185							A	9.72E-04	0.07401
SIP110	1							13A	0.00989	0.16661			
SIP126	3, 4				12A	0.00928	0.11736						
					12A	0.00274	0.04356				A	0.00283	0.02201
SIP152	1–4												
					12A	0.00554	0.03747						
SIP160	3, 4										A/P	0.0061	0.01891
								13A	0.00399	0.06064	A	0.00759	0.01751
SIP161	1–4												
											A/P	0.00773	0.02119
SIP165	1	11 A/P	0.00785	0.10855									
		11A	0.00952	0.10196									
		11A	0.00143	0.15007								3.51	
SIP172	1–4										A		0.03127
					12A	0.00328	0.04192					E-04	
											A		0.03127
					12A	0.00328	0.04192					3.51E-04	
											A	0.00353	0.02108
											A	0.00295	0.02188
SIP182	1–4												
		11A	0.00348	0.12768									
								13 A/P	0.00464	0.05914	A/P	0.00512	0.01966
SIP195	1, 2	11A	8.22E-04	0.16156							A	0.00581	0.01868
					12A	0.00261	0.04474				A	4.78E-04	0.03058
SIP202	1–4										A	0.00176	0.0246
					12 A/P	0.00758	0.03602				A/P	0.00835	0.01792
					12 A/P	4.76E-04	0.06228						
SIP215	1–4												

					12A	0.00133	0.05002				A	6.26E-04	0.03436
					12 A/P	0.00178	0.04746				A/P	0.00149	0.02972
					12A	3.29E-07	0.12172				A	6.94E-06	0.05863
					12 A/P	1.16E-18	0.32087				A/P	1.53E-25	0.27821
SIP220	3, 4										A/P	0.00295	0.0266
												0.00198	0.02885
SIP223	3, 4				12 A/P	0.00622	0.03786						
SIP236	1–4										A	0.00552	0.01909
											A	0.00744	0.01777
SIP237	1	11A	9.97E-04	0.15911	12 A/P	0.00353	0.04314				A	0.00808	0.01745
					12 A/P	0.00153	0.05113						
SIP241	1–4				12 A/P	0.00843	0.03453				A/P	9.10E-04	0.02764
SIP243	1	11A	0.00566	0.11355									
		11A	0.00566	0.11355									
SIP255	1–4							13A	6.54E-04	0.0858	A	0.00475	0.02383
								13A	9.45E-04	0.08095	A	0.00786	0.02115
SIP257	3, 4										A/P	0.0085	0.02085
SIP258	1–4				12 A/P	0.006	0.03843	13A	0.00298	0.06628	A	0.00368	0.02121
SIP265	1–4				12A	3.23E-04	0.063				A	0.00217	0.02829
					12 A/P	0.00656	0.06975						
SIP274	1–4				12 A/P	0.00136	0.05111						
SIP279	3, 4				12 A/P	2.52E-04	0.13251						
SIP297	3, 4				12 A/P	0.00669	0.03696						
