# Supplementary material for: Development and Identification of SSR Markers Associated with Starch Properties and β-Carotene Content in the Storage Root of Sweet Potato (Ipomoea batatas L.)
Source: Front Plant Sci. 2016 Mar 2;7:223. doi: 10.3389/fpls.2016.00223 (PMC4773602; doi:10.3389/fpls.2016.00223)
Supplement: Supplementary Material 12 — Amplicons of SSR markers associated with starch composition shown in Supplementary Material 11. The first lane of each figure is the molecular weight marker, and the next six lanes are amplicons amplified from DNA isolated from the six accessions. Lanes 2–4 (from left to right) are Mianfen No. 1, Nancy Hall, and 0929-106, which had average storage root amylose contents over the 3-year period of 28.465 ± 0.798, 26.081 ± 3.688, and 28.114 ± 1.776%, respectively, and average storage root starch contents over the 3-year period of 30.383 ± 3.713, 19.585 ± 0.231, and 10.857 ± 0.109%, respectively. Lanes 5–7 are Sanheshu, Jishu 52, and Wugonghong, which had average storage root amylose contents over the 3-year period of 15.266 ± 1.656, 13.599 ± 1.206, and 12.076 ± 1.279%, respectively, and average storage root starch contents over the 3-year period of 20.210 ± 2.434, 18.336 ± 1.773, and 11.100 ± 0.824%, respectively. [file DataSheet12.pdf]

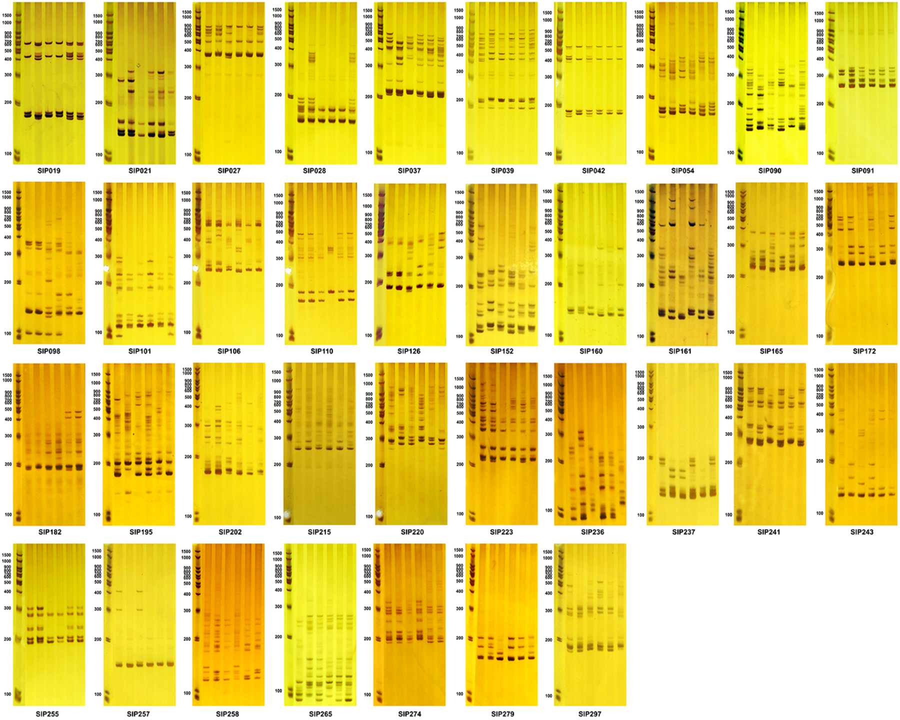

Supplementary Material 12 Amplicons of SSR markers associated with starch composition shown in **Supplementary Material 11**

The first lane of each figure is the molecular weight marker, and the next six lanes are amplicons amplified from DNA isolated from the six accessions. Lanes 2–4 (from left to right) are Mianfen No.1, Nancy Hall, and 0929-106, which had average storage root amylose contents over the 3-year period of 28.465±0.798, 26.081±3.688, and 28.114±1.776%, respectively, and average storage root starch contents over the 3-year period of 30.383±3.713, 19.585±0.231, and 10.857±0.109%, respectively. Lanes 5–7 are Sanheshu, Jishu 52, and Wugonghong, which had average storage root amylose contents over the 3-year period of 15.266±1.656, 13.599±1.206, and 12.076 ±1.279%, respectively, and average storage root starch contents over the 3-year period of 20.210±2.434, 18.336 ±1.773, and 11.100±0.824%, respectively.
