# Supplementary material for: Development and Identification of SSR Markers Associated with Starch Properties and β-Carotene Content in the Storage Root of Sweet Potato (Ipomoea batatas L.)
Source: Front Plant Sci. 2016 Mar 2;7:223. doi: 10.3389/fpls.2016.00223 (PMC4773602; doi:10.3389/fpls.2016.00223)
Supplement: Supplementary Material 13 — Nucleotide sequences of polymorphic bands generated using a primer pair designed to amplify SSR marker SIP183. [file DataSheet13.PDF]

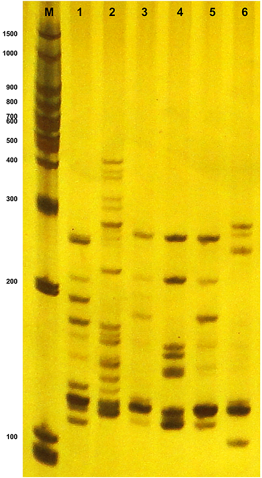

TGCACCCAAGACATCAAAGAAACCCTTCAATTCTCCTCTCTACCAATTCATTTTATGTAATTTCTTCTTCTTCTTCTTCTTCTTCTTCTTCTTCTTCTTCTTCACCATCACTCATTATATTCAACCCAAGATTTTGCTCAG

TGCACCCAAGACATCAAAGAAACCCTTCAATTCTCCTCTCTACCAATTCATTTTATGTAATTTCATCTTCTTCTTCTTCTTCTTCT TCACCATCACTCATTTTATTCAACCCAAGATTTTGCTCAG

TGCACCCAAGACATCAAAGAAACCCTCCAATTCTCCTCTCTACCAATTCATTCTATGTAATTTCATCTTCTTCTTCTTCTTCT TCACCATCACTCATTTTATTCAACCCAAGATTTTGCTCAG

TGCACCCAAGACATCAAAGAAACCCTTCAATTCTCCTCTCTACCAATTCATTTTATGTAATTTCATCTTCTTCTTCT TCACCATCACTCATTATATTCAACCCAAGATTTTGCTCAG

TGCACCCAAGACATCAAAGAAACCCTTCAATTCTCCTCTCTACCAATTCATTTTATGTAATTTCTTCTTCT TCACCATCACTCATTATATTCAACCCAAGATTTTGCTCAG

Supplementary Material 13 Nucleotide sequences of polymorphic bands generated using a primer pair designed to amplify SSR marker SIP183
